# Phenolic acids from medicinal and edible homologous plants: a potential anti-inflammatory agent for inflammatory diseases

**DOI:** 10.3389/fimmu.2024.1345002

**Published:** 2024-06-21

**Authors:** Jingchen Xie, Suhui Xiong, Yamei Li, Bohou Xia, Minjie Li, Zhimin Zhang, Zhe Shi, Qiuxian Peng, Chun Li, Limei Lin, Duanfang Liao

**Affiliations:** ^1^ Key Laboratory for Quality Evaluation of Bulk Herbs of Hunan Province, School of Pharmacy, Hunan University of Chinese Medicine, Changsha, China; ^2^ Institute of Chinese Materia Medica, China Academy of Chinese Medical Sciences, Beijing, China

**Keywords:** medicinal and edible homology, plant sources, structure and distribution, phenolic acids, anti-inflammatory, inflammatory diseases, mechanism, pathway

## Abstract

Inflammation has been shown to trigger a wide range of chronic diseases, particularly inflammatory diseases. As a result, the focus of research has been on anti-inflammatory drugs and foods. In recent years, the field of medicinal and edible homology (MEH) has developed rapidly in both medical and food sciences, with 95% of MEH being associated with plants. Phenolic acids are a crucial group of natural bioactive substances found in medicinal and edible homologous plants (MEHPs). Their anti-inflammatory activity is significant as they play a vital role in treating several inflammatory diseases. These compounds possess enormous potential for developing anti-inflammatory drugs and functional foods. However, their development is far from satisfactory due to their diverse structure and intricate anti-inflammatory mechanisms. In this review, we summarize the various types, structures, and distribution of MEHP phenolic acids that have been identified as of 2023. We also analyze their anti-inflammatory activity and molecular mechanisms in inflammatory diseases through NF-κB, MAPK, NLRP3, Nrf2, TLRs, and IL-17 pathways. Additionally, we investigate their impact on regulating the composition of the gut microbiota and immune responses. This analysis lays the groundwork for further exploration of the anti-inflammatory structure-activity relationship of MEHP phenolic acids, aiming to inspire structural optimization and deepen our understanding of their mechanism, and provides valuable insights for future research and development in this field.

## 1 Introduction

Inflammatory diseases can trigger abnormal reactions in various body systems, leading to tissue damage and dysfunction, which seriously affects human health, and inflammation is the basis of inflammatory diseases. Inflammation is a cascade of chemical signals triggered by viral and bacterial infections, toxic compound stimulation, and tissue damage, which can activate white blood cells to produce and release inflammatory cytokines. Chronic inflammation can contribute to the development of various chronic diseases such as inflammatory diseases, autoimmune diseases, tumors, neurogenic diseases, diabetes, cardiovascular diseases, and tissue fibrosis (1, 2). Therefore, anti-inflammatory drugs and foods have always been a hot topic of research. Medicinal and edible homology (MEH) refers to natural resources offering edible and medicinal value. Being safe and healthy options with medicinal functions, MEH-based research and product development are receiving increasing attention (3, 4). In 2002, a list of items that function as both food and medicine (the catalog of “Medicinal and edible homologous” sources) was released by the former Chinese Ministry of health. As of now, there are 110 Chinese medicinal materials that have been included (3), and 102 of these are plants, accounting for nearly 95%.

Medicinal and edible homologous plants (MEHPs) are characterized by the presence of a variety of active ingredients. Phenolic acids are one of the most representative ingredients of MEHPs. Phenolic acids are a class of organic acids with directly linked phenolic groups to aromatic rings, are abundant in plants, encompassing a broad spectrum of medicinal and edible varieties, and constitute vital secondary metabolites. Currently, phenolic acids hold broad applications across various sectors, including the food industry, medicine, health supplements, and cosmetics. They are integrated into food products as natural preservatives and antioxidants, enhancing shelf life (5). Within the pharmaceutical domain, phenolic acids serve as therapeutic agents or adjuvants for combating inflammatory conditions and select cancers (6). As dietary supplements, they contribute to health promotion and disease prevention (7). Additionally, phenolic acids are leveraged in skincare for their potent antioxidant and anti-inflammatory benefits, particularly in anti-aging and protective formulations (8). The structural skeleton of phenolic acids is mainly composed of a carboxyl group and one or more hydroxyl groups bound to aromatic rings. Phenolic acids can be divided into three classes: hydroxybenzoic acids, hydroxyphenylacetic acids, and hydroxycinnamic acids, all having anti-inflammatory, anti-oxidant, anti-bacterial, and anti-viral activities (9–12). Bioactivity is closely related to the structure of MEHP phenolic acids; hence, an understanding of the varied structures of these compounds is important.

Anti-inflammatory activity is one of the main features of MEHP phenolic acids and plays an important role in the prevention and treatment of numerous inflammatory diseases (13–16). Although the pathogenesis of these diseases is different, the regulation of inflammatory signaling pathways is similar. Therefore, it is essential to elucidate the anti-inflammatory mechanisms of MEHP phenolic acids for intensive research on their anti-inflammatory diseases’ activity.

We performed a comprehensive database search of PubMed, Web of Science, and Science Direct for entries up to November 2023, to systematically review the types, structures, anti-inflammatory activities, and molecular mechanisms of MEHP phenolic acids. The objective is to provide scientific basis for in-depth research and comprehensive development of the anti-inflammatory activities of MEHP phenolic acids.

## 2 Structure and distribution of MEHP phenolic acids

Upon conducting a thorough literature review, we discovered that 68 types of MEHP were reported to contain a comprehensive collection of 167 phenolic acids. Among these, there are 45 hydroxybenzoic acids, 113 hydroxycinnamic acids, 8 hydroxyphenylacetic acids, and 1 other phenolic acid. The 68 MEHPs belong to 35 families with 6 species from Rosaceae or Lamiaceae, 5 from Zingiberaceae, 4 from Caprifoliaceae or Compositae, and 3 from Moraceae, Rutaceae, Leguminosae, or Campanulacea.

### 2.1 Hydroxybenzoic acids

**Figure 1:**
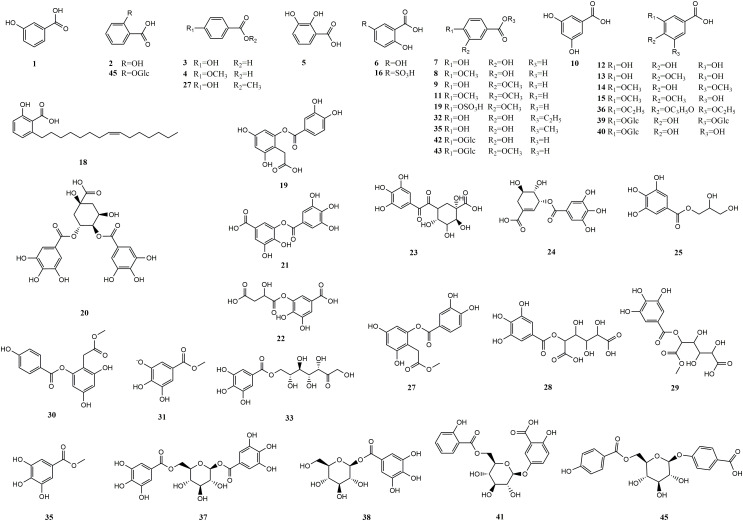
Structures of hydroxybenzoic acids from medicinal and edible homologous plants.

Hydroxybenzoic acids are based on a hydroxybenzoic acid skeleton. The hydroxybenzoic acids can be divided into simple hydroxybenzoic acids, polyhydroxybenzoic acids, hydroxybenzoates, and hydroxybenzoate glycosides. According to reports, there are 18 types of simple hydroxybenzoic acids, 6 types of polyhydroxybenzoic acids, 12 types of hydroxybenzoates, and 9 types of hydroxybenzoate glycosides. Simple hydroxybenzoic acids are the most widely distributed (including vanillic acid, gallic acid, syringic acid, salicylic acid, protocatechuic acid, p-hydroxybenzoic acid, etc.) among which vanillic acid, gallic acid, and syringic acid are distributed in 28, 25, and 24 MEHPs, respectively. Details are shown in **Table 1** and the structure is shown in **Figure 1**.

**Table 1:** Hydroxybenzoic acids of medicinal and edible homologous plants.

No.	Components	Molecular Formula	MEHPs
Simple hydroxybenzoic acids			
**1**	3-hydroxybenzoic acid	C_7_H_6_O_3_	*Lycium barbarum* L (17); *Sesamum indicum* L (18); *Crocus sativus* L (19); *Amomum tsao-ko Crevost* et Lemaire (20).
**2**	salicylic acid	C_7_H_6_O_3_	*Cichorium intybus* L (21); *Hippophae rhamnoides* L (22); *Perilla frutescens* (L.) Britt. (leaf) (23); *Sesamum indicum* L (18); *Panax ginseng* C.A.Mey (24); *Crocus sativus* L (25); *Curcuma longa* L (26); *Panax quinquefolium* L (27).
**3**	p-hydroxybenzoic acid	C_7_H_6_O_3_	*Hippophae rhamnoides* L (28); *Hordeum vulgare* L (29); *Laminaria japonica* Aresch (30); *Houttuynia cordata* Thunb (31); *Zingiber officinale* Rosc (32); *Lycium barbarum* L (17); *Sterculia lychnophora* Hance (33); *Morus alba* L. (fruit) (34); *Nelumbo nucifera* Gaertn. (fruit) (35); *Nelumbo nucifera* Gaertn. (leaf) (36); *Cichorium intybus* L (37); *Perilla frutescens* (L.) Britt. (leaf) (38); *Sesamum indicum* L (18); *Angelica sinensis* (Oliv.) Diels (39); *Kaempferia galanga* L (40); *Crocus sativus* L (41); *Panax quinquefolium* L (27); *Gastrodia elata* B1 (42); *Piper nigrum* L (43); *Panax ginseng* C.A.Mey (44); *Coriandrum sativum* L (45).
**4**	anisic acid	C_8_H_8_O_3_	*Kaempferia galanga* L (40).
**5**	pyrocatechuic acid	C_7_H_6_O_4_	*Hippophae rhamnoides* L (28); *Hordeum vulgare* L (46).
**6**	gentisic acid	C_7_H_6_O_4_	*Hippophae rhamnoides* L (28); *Nelumbo nucifera* Gaertn. (fruit) (47); *Dimocarpus longan* Lour (48); *Panax ginseng* C.A.Mey (44); *Rosa rugosa* Thunb (49); *Crocus sativus* L (25).
**7**	protocatechuic acid	C_7_H_6_O_4_	*Hippophae rhamnoides* L (28); *Hordeum vulgare* L (46); *Ziziphus jujuba* Mill (50); *Lycium barbarum* L (17); *Gardenia jasminoides* Ellis (51); *Sterculia lychnophora* Hance (33); *Mosla chinensis* ‘jiangxiangru’ (52); *Morus alba* L. (fruit) (34); *Morus alba* L. (leaf) (53); *Alpinia oxyphylla* Miq (54); *Nelumbo nucifera* Gaertn. (fruit) (35); *Nelumbo nucifera* Gaertn. (leaf) (36); *Perilla frutescens* (L.) Britt. (leaf) (38); *Piper nigrum* L (43); *Panax ginseng* C.A.Mey (44); *Rosa rugosa* Thunb (49); *Prunella vulgaris* L (55); *Angelica sinensis* (Oliv.) Diels (39); *Panax quinquefolium* L (27);*Cornus officinalis* Sieb. et Zucc (56); *Eucommia ulmoides* Oliv (57).
**8**	isovanillic acid	C_8_H_8_O_4_	*Vigna umbellata* Ohwi et Ohashi (58); *Vigna angularis* Ohwi et Ohashi (58); *Perilla frutescens* (L.) Britt. (Leaf) (38).
**9**	vanillic acid	C_8_H_8_O_4_	*Crataegus pinnatifida* Bge (59); *Dimocarpus longan* Lour (48); *Hippophae rhamnoides* L (60); *Vigna umbellata* Ohwi et Ohashi (58); *Vigna angularis* Ohwi et Ohashi (58); *Hordeum vulgare* L (29); *Laminaria japonica* Aresch (30); *Houttuynia cordata* Thunb (31); *Hovenia dulcis* Thunb (61); *Lycium barbarum* L (17); *Morus alba* L. (fruit) (34); *Platycodon grandiflorum* (Jacq.) A.DC (62); *Nelumbo nucifera* Gaertn. (leaf) (36); *Cichorium intybus* L (21); *Perilla frutescens*(L.) Britt. (Leaf) (63); *Perilla frutescens* (L.) Britt. (fruit) (64); *Sesamum indicum* L (18); *Panax ginseng* C.A.Mey (65); *Coriandrum sativum* L (45); *Angelica sinensis* (Oliv.) Diels (39); *Kaempferia galanga* L (40); *Crocus sativus* L (41); *Curcuma Longa* L (26); *Codonopsis pilosula* (Franch.) Nannf (66); *Dendrobium officinale* Kimura et Migo (67); *Panax quinquefolium* L (27); *Gastrodia elata* B1 (68); *Dolichos lablab* L (69).
**10**	3,5-dihydroxybenzoic acid	C_7_H_6_O_4_	*Amomum tsao-ko Crevost* et Lemaire (20).
**11**	veratric acid	C_9_H_10_O_4_	*Hippophae rhamnoides* L (28).
**12**	gallic acid	C_7_H_6_O_5_	*Portulaca oleracea* L (70); *Dolichos lablab* L (69); *Dimocarpus longan Lour (* 48 *).*; *Phyllanthus emblica* L (71); *Citrus medica* L (72); *Hippophae rhamnoides* L (60); *Vigna umbellata* Ohwi et Ohashi (58); *Vigna angularis* Ohwi et Ohashi (58); *Hordeum vulgare* L (46); *Laminaria japonica* Aresch (30); *Ziziphus jujuba* Mill (73); *Canarium album* Raeusch (72); *Houttuynia cordata* Thunb (31); *Zingiber officinale* Rosc (32); *Lycium barbarum* L (74); *Morus alba* L. (fruit) (75); *Morus alba* L. (leaf) (76); *Citrus reticulata* Blanco (77); *Nelumbo nucifera* Gaertn. (fruit) (47); *Nelumbo nucifera* Gaertn. (leaf) (36); *Perilla frutescens* (L.) Britt. (Leaf) (23); *Panax ginseng* C.A.Mey (78); *Rosa rugosa* Thunb (49); *Crocus sativus* L (41); *Curcuma Longa* L (26); *Panax quinquefolium* L (27); *Cornus officinalis* Sieb. et Zucc (79).
**13**	4-O-methylgallic acid	C_8_H_8_O_5_	*Phyllanthus emblica* L (71); *Piper nigrum* L (43).
**14**	syringic acid	C_9_H_10_O_5_	*Portulaca oleracea* L (80); *Cannabis sativa* L (81); *Dolichos lablab* L (69); *Dimocarpus longan* Lour (48); *Phyllanthus emblica* L (71); *Hordeum vulgare* L (29); *Houttuynia cordata* Thunb (31); *Zingiber officinale* Rosc (32); *Lycium barbarum* L (74); *Morus alba* L. (fruit) (82); *Citrus reticulata* Blanco (77); *Nelumbo nucifera* Gaertn. (leaf) (36); *Perilla frutescens*(L.) Britt. (Leaf) (63); *Sesamum indicum* L (18); *Piper nigrum* L (43); *Panax ginseng* C.A.Mey (44); *Coriandrum sativum* L (45); *Angelica sinensis* (Oliv.) Diels (83); *Crocus sativus* L (25); *Curcuma longa* L (26); *Dendrobium officinale* Kimura et Migo (67); *Panax quinquefolium* L (27).
**15**	3,4-O-dimethylgallic acid	C_9_H_10_O_5_	*Piper nigrum* L (43).
**16**	5-sulfosalicyclic acid	C_7_H_6_O_6_S	*Perilla frutescens* (L.) Britt. (Leaf) (63)
**17**	vanillic acid 4-sulfate	C_8_H_8_O_7_S	*Piper nigrum* L (43).
**18**	ginkgolic acid	C_22_H_34_O_3_	*Cistanche deserticola* Y.C.Ma (84).
Polyhydroxybenzoic acids			
**19**	2-O-(3,4-dihydroxybenzoyl)-2,4,6-trihydroxy-phenylacetic acid	C_15_H_12_O_8_	*Morus alba* L. (fruit) (85)
**20**	3,4-di-O-galloylquinic acid	C_21_H_20_O_14_	*Phyllanthus emblica* L (71).
**21**	digallic acid	C_14_H_10_O_9_	*Canarium album* Raeusch (86).
**22**	gallic acid O-malic acid	C_10_H_10_O_9_	*Canarium album* Raeusch (86).
**23**	galloylquinic acid	C_14_H_16_O_10_	*Canarium album* Raeusch (86).
**24**	galloylshikimic acid	C_14_H_14_O_9_	*Canarium album* Raeusch (86).
Hydroxybenzoates			
**25**	1-O-galloyl-glycerol	C_10_H_12_O_7_	*Phyllanthus emblica* L (71).
**26**	methylparaben	C_8_H_8_O_3_	*Crocus sativus* L (41).
**27**	2-O-(3,4-dihydroxybenzoyl)-2,4,6-trihydroxyphenylmethylacetate	C_16_H_14_O_8_	*Morus alba* L. (fruit) (85)
**28**	2-O-galloylgalactaric acid	C_13_H_14_O_12_	*Phyllanthus emblica* L (71).
**29**	1-methyl 2-galloylgalactarate	C_14_H_15_O_12_	*Phyllanthus emblica* L (71).
**30**	3,5-dihydroxy-2-(2-methoxy-2-oxoethyl) phenyl 4-hydroxybenzoate	C_16_H_14_O_7_	*Cornus officinalis* Sieb. et Zucc (56)
**31**	3-O-methylgallate	C_8_H_7_O_5_	*Phyllanthus emblica* L (71).
**32**	protocatechuic acid ethyl ester	C_9_H_10_O_4_	*Sterculia lychnophora* Hance (33); *Morus alba* L. (fruit) (85)
**33**	7-O-galloyl-d-sedoheptulose	C_14_H_18_O_11_	*Cornus officinalis* Sieb. et Zucc (79)
**34**	protocatechuic acid methyl ester	C_8_H_8_O_4_	*Kaempferia galanga* L (40); *Morus alba* L. (fruit) (85)
**35**	methyl gallate	C_8_H_8_O_5_	*Cistanche deserticola* Y.C.Ma (84)
**36**	O-acetylsyringic acid	C_14_H_18_O_6_	*Morus alba* L. (fruit) (82)
Hydroxybenzoate glycosides			
**37**	1-O,6-O-digalloyl-*β*-D-glucose	C_20_H_20_O_14_	*Phyllanthus emblica* L (71).
**38**	*β*-glucogallin	C_13_H_16_O_10_	*Phyllanthus emblica* L (71).
**39**	gallic acid-3,5-diglucoside	C_19_H_26_O_15_	*Angelica sinensis* (Oliv.) Diels (83).
**40**	galloyl-glucoside	C_13_H_16_O_10_	*Angelica sinensis* (Oliv.) Diels (83).
**41**	gentisic acid 5-O-D-(6’-salicyly1)-glucopyranoside	C_20_H_20_O_11_	*Prunella vulgaris* L (87).
**42**	protocatechuic acid 4-O-glucoside	C_13_H_16_O_9_	*Piper nigrum* L (43).
**43**	vanillic acid -4-O-glucoside	C_14_H_18_O_9_	*Mosla chinensis* ‘jiangxiangru’ (52); *Sesamum indicum* L (18).
**44**	salicylic acid-2-O-glucoside	C_13_H_16_O_8_	*Sesamum indicum* L (18).
**45**	1-O-4-carboxylphenyl-(6-O-4-hydroxybenzoyl)-*β*-D-glucopyranoside	C_20_H_20_O_10_	*Kaempferia galanga* L (40).

### 2.2 Hydroxycinnamic acids

**Figure 2:**
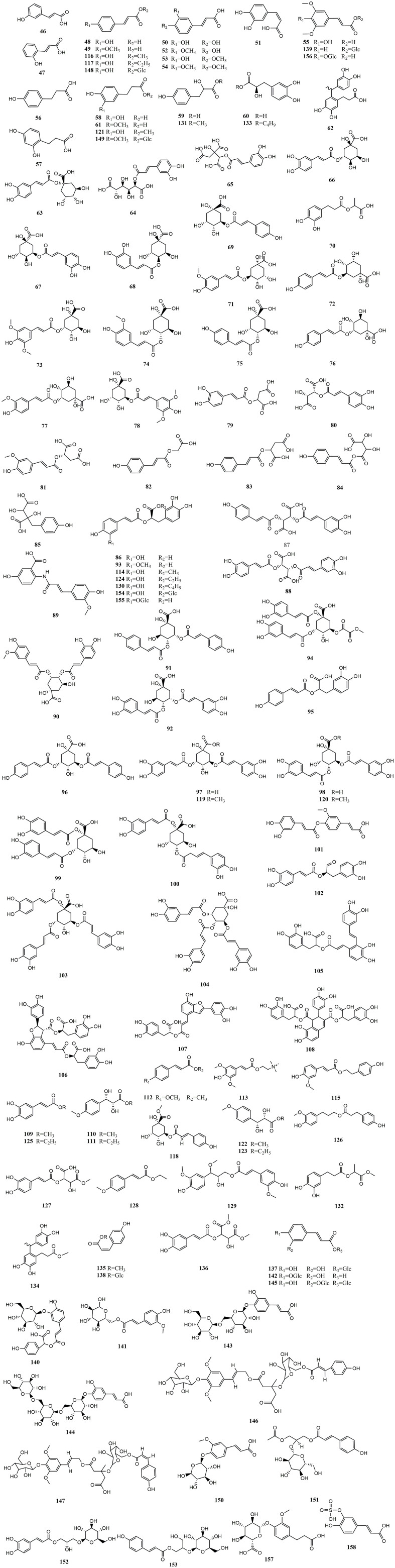
Structures of hydroxycinnamic acids from medicinal and edible homologous plants.

Hydroxycinnamic acids are the most abundant and widely distributed phenolic acids. According to structure, they can be divided into simple hydroxycinnamic acids, hydrogenated hydroxycinnamic acids, polyhydroxycinnamic acids, hydroxycinnamates, hydroxycinnamate glycosides, and hydroxycinnamate salts. Among the reported MEHP phenolic acids, there are 10 simple hydroxycinnamic acids, 7 hydrogenated hydroxycinnamic acids, 46 polyhydroxycinnamic acids, 26 hydroxycinnamates, 21 hydroxycinnamate glycosides, and 1 hydroxycinnamate salt. Among these, simple hydroxycinnamic acids and polyhydroxycinnamic acids are the most diverse. The most widely distributed simple hydroxycinnamic acids include caffeic acid, ferulic acid, and p-coumaric acid, which are distributed in 39, 31, and 28 MEHPs, respectively. Most polyhydroxycinnamic acids have caffeic acid as the parent core, including caffeoylquinic acids which combine caffeic acid and quinic acid (chlorogenic acid) and rosmarinic acid which is a combination of caffeic acid and danshensu. Chlorogenic acid is the phenolic acid with the largest reported distribution in 43 MEHPs. In addition, the reported hydroxycinnamate salt (caffeic acid 3-sulfonate) was only distributed in Piper nigrum L. Detailed information is shown in **Table 2** and the structure is shown in **Figure 2**.

**Table 2:** Hydroxycinnamic acids of medicinal and edible homologous plants.

No.	Components	Molecular Formula	MEHPs
Simple hydroxycinnamic acids			
**46**	m-coumaric acid	C_9_H_8_O_3_	*Hippophae rhamnoides* L (28); *Piper nigrum* L (43); *Panax ginseng* C.A.Mey (44); *Morus alba* L. (fruit) (34)
**47**	o-coumaric acid	C_9_H_8_O_3_	*Morus alba* L. (fruit) (88); *Perilla frutescens* (L.) Britt. (leaf) (63); *Panax ginseng* C.A.Mey (44); *Crocus sativus* L (19).
**48**	p-coumaric acid	C_9_H_8_O_3_	*Cornus officinalis* Sieb.et Zucc (79); *Portulaca oleracea* L (70); *Prunella vulgaris* L (87); *Prunus mume* (Sieb.) Sieb. et Zucc (89); *Dolichos lablab* L (69); *Dendrobium officinale* Kimura et Migo (67); *Dimocarpus longan* Lour (48); *Citrus medica* L (72); *Hordeum vulgare* L (29); *Houttuynia cordata* Thunb (31); *Zingiber officinale* Rosc (32); *Lycium barbarum* L (17); *Morus alba* L. (fruit) (34); *Morus alba* L. (leaf) (90); *Alpinia oxyphylla* Miq (91); *Nelumbo nucifera* Gaertn. (fruit) (47); *Nelumbo nucifera* Gaertn. (leaf) (36); *Chrysanthemum morifolium* Ramat (92); *Cichorium intybus* L (37); *Rubus chingii* Hu (93); *Panax ginseng* C.A.Mey (44); *Coriandrum sativum* L (94); *Prunella vulgaris* L (95); *Crocus sativus* L (41); *Curcuma longa* L (26); *Panax quinquefolium* L (27); *Hippophae rhamnoides* L (96); *Kaempferia galanga* L (40).
**49**	trans p-methoxycinnamic acid	C_10_H_10_O_3_	*Kaempferia galanga* L (40).
**50**	caffeic acid	C_9_H_8_O_4_	*Cirsium setosum* (Willd.) MB (97); *Portulaca oleracea* L (70); *Prunus mume* (Sieb.) Sieb. et Zucc (89); *Phyllanthus emblica* L (71); *Citrus medica* L (72); *Hippophae rhamnoides* L (98); *Vigna umbellata* Ohwi et Ohashi (58); *Vigna angularis* Ohwi et Ohashi (58); *Laminaria japonica* Aresch (30); *Ziziphus jujuba* Mill (73); *Lonicera japonica* Thunb (99); *Zingiber officinale* Rosc (32); *Lycium barbarum* L (17); *Sterculia lychnophora* Hance (33); *Morus alba* L. (fruit) (34); *Morus alba* L. (leaf) (53); *Citrus reticulata* Blanco (100); *Alpinia oxyphylla* Miq (91); *Nelumbo nucifera* Gaertn. (fruit) (47); *Nelumbo nucifera* Gaertn. (leaf) (36); *Lophatherum gracile* Brongn (101); *Chrysanthemum morifolium* Ramat (102); *Perilla frutescens* (L.) Britt. (leaf) (103); *Perilla frutescens* (L.) Britt. (fruit) (64); *Sesamum indicum* L (18); *Piper nigrum* L (43); *Taraxacum mongolicum* Hand.-Mazz (104); *Mentha haplocalyx* Briq (105); *Panax ginseng* C.A.Mey (78); *Lonicera hypoglauca* Miq (106); *Lonicera macranthoides* Hand.-Mazz (107); *Coriandrum sativum* L (45); *Prunella vulgaris* L (108); *Angelica sinensis* (Oliv.) Diels (83); *Crocus sativus* L (41); *Curcuma Longa* L (26); *Codonopsis pilosula* (Franch.) Nannf (66); *Cornus officinalis* Sieb. et Zucc (79); *Eucommia ulmoides* Oliv (57).
**51**	Z-caffeic acid	C_9_H_8_O_4_	*Sterculia lychnophora* Hance (33)
**52**	ferulic acid	C_10_H_10_O_4_	*Prunus mume* (Sieb.) Sieb. et Zucc (89); *Cannabis sativa* L (109); *Dolichos lablab* L (69); *Dimocarpus longan* Lour (48); *Hippophae rhamnoides* L (60); *Vigna umbellata* Ohwi et Ohashi (58); *Vigna angularis* Ohwi et Ohashi (58); *Hordeum vulgare* L (29); *Laminaria japonica* Aresch (30); *Ziziphus jujuba* Mill (73); *Houttuynia cordata* Thunb (31); *Zingiber officinale* Rosc (32); *Hovenia dulcis* Thunb (61); *Lycium barbarum* L (17); *Morus alba* L. (fruit) (34); *Morus alba* L. (leaf) (76); *Citrus reticulata* Blanco (100); *Alpinia oxyphylla* Miq (91); *Nelumbo nucifera* Gaertn. (fruit) (47); *Nelumbo nucifera* Gaertn. (leaf) (36); *Perilla frutescens* (L.) Britt. (leaf) (38); *Sesamum indicum* L (18); *Panax ginseng* C.A.Mey (44); *Lonicera hypoglauca* Miq (110); *Coriandrum sativum* L (45); *Prunella vulgaris* L (55); *Angelica sinensis* (Oliv.) Diels (39); *Kaempferia galanga* L (40); *Curcuma Longa* L (26); *Dendrobium officinale* Kimura et Migo (67); *Panax quinquefolium* L (27).
**53**	(E)-isoferulic acid	C_10_H_10_O_4_	*Panax quinquefolium* L (27)
**54**	3,4-dimethoxycinnamic acid	C_11_H_12_O_4_	*Sesamum indicum* L (18).
**55**	sinapinic acid	C_11_H_12_O_5_	*Portulaca oleracea* L (80); *Dimocarpus longan* Lour (48); *Hordeum vulgare* L (29); *Morus alba* L. (fruit) (82); *Alpinia oxyphylla* Miq (91); *Nelumbo nucifera* Gaertn. (leaf) (36); *Brassica juncea* (L.) Czern.et Coss (111); *Perilla frutescens* (L.) Britt. (leaf) (38); *Sesamum indicum* L (18); *Crocus sativus* L (41); *Curcuma longa* L (26); *Houttuynia cordata* Thunb (31); *Morus alba* L.(leaf) (76)
Hydrogenated hydroxycinnamic acids			
**56**	p-hydroxyphenylpropionic acid	C_9_H_10_O_3_	*Mentha haplocalyx* Briq (112).
**57**	3-(2,4-dihydroxyphenyl) propionic acid	C_9_H_10_O_4_	*Lycium barbarum* L (17).
**58**	dihydrocaffeic acid	C_9_H_10_O_4_	*Eucommia ulmoides* Oliv (57); *Prunella vulgaris* L (113).
**59**	p-hydroxyphenyl-lactic	C_9_H_10_O_4_	*Hippophae rhamnoides* L (28); *Angelica sinensis* (Oliv.) Diels *(* 83 *)*; *Dendrobium officinale* Kimura et Migo (114)
**60**	danshensu	C_9_H_10_O_5_	*Mentha haplocalyx* Briq (105); *Prunella vulgaris* L (113).
**61**	dihydroferulic acid	C_10_H_12_O_4_	*Prunella vulgaris* L (113); *Panax quinquefolium* L (27).
**62**	(±)3-{2-[1-(3′,4′-dihydroxy-phenyl)ethyl]-4,5-dihydroxyphenyl} propanoic acid	C_17_H_18_O_6_	*Eucommia ulmoides* Oliv (57).
Polyhydroxycinnamic acids			
**63**	1-O-caffeoylquinic acid	C_16_H_18_O_9_	*Chrysanthemum morifolium* Ramat (115); *Morus alba* L. (leaf) (116)
**64**	2-O-caffeoylglucarate	C_15_H_16_O_11_	*Phyllanthus emblica* L (71).
**65**	2-O-caffeoylhydroxycitric acid	C_15_H_14_O_11_	*Phyllanthus emblica* L (71).
**66**	chlorogenic acid	C_16_H_18_O_9_	*Cirsium setosum* (Willd.) MB (97); *Crataegus pinnatifida* Bge (59); *Portulaca oleracea* L (70); *Prunus mume* (Sieb.) Sieb. et Zucc (89); *Chaenomeles speciosa* (Sweet) Nakai (117); *Dimocarpus longan* Lour (48); *Cinnamomum cassia* Presl (118); *Phyllanthus emblica* L (71); *Citrus medica* L (72); *Prunus armeniaca* L (119); *Hippophae rhamnoides* L (98); *Zanthoxylum bungeanum* Maxim (120); *Vigna umbellata* Ohwi et Ohashi (58); *Vigna angularis* Ohwi et Ohashi (58); *Ziziphus jujuba* Mill (73); *Lonicera japonica* Thunb (99); *Houttuynia cordata* Thunb (121); *Lycium barbarum* L (74); *Gardenia jasminoides* Ellis (51); *Morus alba* L. (fruit) (34); *Morus alba* L. (leaf) (53); *Citrus reticulata* Blanco (77); *Alpinia oxyphylla* Miq (91); *Nelumbo nucifera* Gaertn. (fruit) (47); *Nelumbo nucifera* Gaertn. (leaf) (36); *Lophatherum gracile* Brongn (122); *Chrysanthemum morifolium* Ramat (102); *Cichorium intybus* L (123); *Perilla frutescens* (L.) Britt. (leaf) (63); *Sesamum indicum* L (18); *Piper nigrum* L (43); *Taraxacum mongolicum* Hand.-Mazz (104); *Mentha haplocalyx* Briq (105); *Panax ginseng* C.A.Mey (44); *Lonicera hypoglauca* Miq (110); *Lonicera macranthoides* Hand.-Mazz (107); *Lonicera fulvotomentosa* Hsu et S.C.Cheng; *Coriandrum sativum* L (124); *Prunella vulgaris* L (55); *Angelica sinensis* (Oliv.) Diels (83); *Crocus sativus* L (41); *Astragalus membranaceus* (Fisch.) Bge.var.mongholicus (Bge.) Hsiao (125); *Eucommia ulmoides* Oliv (57); *Codonopsis pilosula* (Franch.) Nannf (66).
**67**	neochlorogenic acid	C_16_H_18_O_9_	*Crataegus pinnatifida* Bge (59); *Prunus mume* (Sieb.) Sieb. et Zucc (89); *Gardenia jasminoides* Ellis (51); *Mosla chinensis* ‘jiangxiangru’ (52); *Morus alba* L. (fruit) (34); *Morus alba* L. (leaf) (90); *Lophatherum gracile* Brongn (122); *Chrysanthemum morifolium* Ramat (102); *Cichorium intybus* L (37); *Lonicera fulvotomentosa* Hsu et S.C.Cheng (124); *Angelica sinensis* (Oliv.) Diels (83); *Lonicera japonica* Thunb (99).
**68**	cryptochlorogenic acid	C_16_H_18_O_9_	*Lonicera hypoglauca* Miq (106); *Morus alba* L. (fruit) (126); *Lonicera japonica* Thunb (99); *Morus alba* L. (leaf) (90); *Chrysanthemum morifolium* Ramat (102); *Cichorium intybus* L (123); *Sesamum indicum* L (18); *Angelica sinensis* (Oliv.) Diels (83).
**69**	p-coumaroyl quinic acid	C_16_H_18_O_8_	*Alpinia oxyphylla* Miq (91).
**70**	2-{[3-(3,4-dihydroxyphenyl)propanoyl]oxy} propanoic acid	C_12_H_14_O_6_	*Eucommia ulmoides* Oliv (57).
**71**	3-O-feruloylquinic acid	C_17_H_20_O_9_	*Lophatherum gracile* Brongn (101).
**72**	3-O-coumaroylquinic acid	C_16_H_18_O_8_	*Lophatherum gracile* Brongn (122); *Sesamum indicum* L (18); *Alpinia oxyphylla* Miq (91).
**73**	3-O-sinapoylquinic acid	C_18_H_22_O_10_	*Piper nigrum* L (43).
**74**	4-O-feruloylquinic acid	C_17_H_20_O_9_	*Lophatherum gracile* Brongn (101); *Cichorium intybus* L (37).
**75**	4-O-coumaroylquinic acid	C_16_H_18_O_8_	*Lophatherum gracile* Brongn (122); *Sesamum indicum* L (18).
**76**	5-O-coumaroylquinic acid	C_16_H_18_O_8_	*Alpinia oxyphylla* Miq (91); *Lophatherum gracile* Brongn (122); *Sesamum indicum* L (18).
**77**	5-O-feruloylquinic acid	C_17_H_20_O_9_	*Sesamum indicum* L (18).
**78**	5-O-sinapoylquinic acid	C_18_H_22_O_10_	*Chrysanthemum morifolium* Ramat (115).
**79**	caffeoylmalic acid	C_13_H_12_O_8_	*Phyllanthus emblica* L (71).
**80**	caftaric acid	C_13_H_12_O_9_	*Phyllanthus emblica* L (71); *Cichorium intybus* L (123); *Taraxacum mongolicum* Hand.-Mazz (104).
**81**	feruloyl tartaric acid	C_14_H_14_O_8_	*Piper nigrum* L (43).
**82**	p-coumaroyl glycolic acid	C_11_H_10_O_5_	*Piper nigrum* L (43).
**83**	p-coumaroyl malic acid	C_13_H_12_O_7_	*Piper nigrum* L (43); *Alpinia oxyphylla* Miq (91).
**84**	P-coumaroyl tartaric acid	C_13_H_12_O_8_	*Perilla frutescens* (L.) Britt. (leaf) (127); *Piper nigrum* L (43).
**85**	piscidic acid	C_11_H_12_O_7_	*Angelica sinensis* (Oliv.) Diels (83).
**86**	rosmarinic acid	C_18_H_16_O_8_	*Vigna umbellata* Ohwi et Ohashi (58); *Mosla chinensis* ‘jiangxiangru’ (52); *Morus alba* L. (leaf) (76); *Nelumbo nucifera* Gaertn. (fruit) (47); *Perilla frutescens* (L.) Britt. (leaf) (103); *Perilla frutescens* (L.) Britt. (fruit) (64); *Piper nigrum* L (43); *Pogostemon cablin* (Blanco) Benth (128); *Coriandrum sativum* L (45); *Prunella vulgaris* L (108).
**87**	p-coumaroylcaffeoyltartaric acid	C_22_H_18_O_11_	*Sesamum indicum* L (18).
**88**	cichoric acid	C_22_H_18_O_12_	*Cichorium intybus* L (123); *Taraxacum mongolicum* Hand. Mazz (104).
**89**	avenanthramide 2f	C_17_H_15_NO_6_	*Piper nigrum* L (43).
**90**	4-O-caffeoyl-5-O-feruloylquinic acid	C_26_H_26_O_12_	*Chrysanthemum morifolium* Ramat (115).
**91**	4,5-di-O-p-coumaroylquinic acid	C_25_H_24_O_10_	*Lonicera hypoglauca* Miq (106).
**92**	4,5-di-O-caffeoylquinic acid	C_25_H_24_O_12_	*Chrysanthemum morifolium* Ramat (102); *Cichorium intybus* L (129).
**93**	3-O-methylrosmarinic acid	C_19_H_18_O_8_	*Piper nigrum* L (43).
**94**	3-O-methoxyoxaloyl-1,5-di-O-caffeoylquinic acid	C_28_H_26_O_15_	*Chrysanthemum morifolium* Ramat (115).
**95**	3′-dehydroxylation rosmarinic acid	C_18_H_16_O_7_	*Perilla frutescens* (L.) Britt. (leaf) (130)
**96**	3,5-di-O-p-coumaroylquinic acid	C_24_H_23_O_10_	*Lonicera hypoglauca* Miq (106).
**97**	3,5-di-O-caffeoylquinic acid	C_25_H_24_O_12_	*Lonicera japonica* Thunb (99); *Morus alba* L. (fruit) (126); *Chrysanthemum morifolium* Ramat (102); *Cichorium intybus* L (129); *Taraxacum mongolicum* Hand.-Mazz (104); *Lonicera fulvotomentosa* Hsu et S.C.Cheng (131); *Morus alba* L. (leaf) (53)
**98**	3,4-di-O-caffeoylquinic acid	C_25_H_24_O_12_	*Lonicera japonica* Thunb (99); *Chrysanthemum morifolium* Ramat (102); *Lonicera fulvotomentosa* Hsu et S.C.Cheng (131); *Gardenia jasminoides* Ellis (132)
**99**	1,5-di-O-caffeoylquinic acid	C_25_H_24_O_12_	*Morus alba* L. (fruit) (82); *Morus alba* L. (leaf) (53)
**100**	1,4-di-O-caffeoylquinic acid	C_25_H_24_O_12_	*Lonicera japonica* Thunb (133).
**101**	caffeoyl-ferulic acid	C_19_H_16_O_7_	*Morus alba* L. (fruit) (82)
**102**	rosmarinic acid decarboxylation	C_18_H_16_O_7_	*Prunella vulgaris* L (113).
**103**	1,3,5-tricaffeoylquinic acid	C_34_H_30_O_15_	*Morus alba* L. (leaf) (134)
**104**	3,4,5-tricaffeoylquinic acid	C_34_H_30_O_15_	*Morus alba* L. (leaf) (134); *Chrysanthemum morifolium* Ramat (115).
**105**	salvianolic acid A	C_26_H_22_O_10_	*Angelica sinensis* (Oliv.) Diels (83).
**106**	salvianolic acid B	C_36_H_30_O_16_	*Angelica sinensis* (Oliv.) Diels (83); *Mentha haplocalyx* Briq (105).
**107**	salvianolic acid C	C_26_H_20_O_10_	*Angelica sinensis* (Oliv.) Diels (83).
**108**	salvianolic acid L	C_36_H_30_O_16_	*Angelica sinensis* (Oliv.) Diels (83); *Mentha haplocalyx* Briq (105).
Hydroxycinnamates			
**109**	methyl caffeate	C_10_H_10_O_4_	*Prunella vulgaris* L (113).
**110**	methyl (2R,3S)-2,3-dihydroxy-3-(4-methoxyphenyl) propanoate	C_11_H_14_O_6_	*Kaempferia galanga* L (40).
**111**	ethyl (2R,3S)-2,3-dihydroxy-3-(4-methoxyphenyl) propanoate	C_12_H_16_O_6_	*Kaempferia galanga* L (40).
**112**	trans ethyl p-methoxycinnamate	C_11_H_12_O_3_	*Kaempferia galanga* L (40).
**113**	sinapine	C_16_H_24_NO_5_	*Brassica juncea* (L.) Czern.et Coss (111)
**114**	methyl rosmarinate	C_19_H_18_O_8_	*Perilla frutescens* (L.) Britt. (fruit) (64); *Perilla frutescens* (L.) Britt. (leaf) (103); *Prunella vulgaris* L (113).
**115**	p-hydroxyphenethyl trans-ferulate	C_18_H_18_O_5_	*Angelica sinensis* (Oliv.) Diels (135).
**116**	p-coumaric acid methyl este	C_10_H_10_O_3_	*Sesamum indicum* L (18); *Cannabis sativa* L (109).
**117**	p-coumaric acid ethyl ester	C_11_H_12_O_3_	*Sesamum indicum* L (18).
**118**	methyl coumaroyl quinic acid	C_17_H_20_O_8_	*Morus alba* L. (fruit) (82)
**119**	methyl 3,5-di-O-caffeoylquinate	C_26_H_26_O_12_	*Lonicera fulvotomentosa* Hsu et S.C.Cheng (131); *Lonicera japonica* Thunb (133).
**120**	methyl 3,4-di-O-caffeoylquinate	C_26_H_26_O_12_	*Lonicera fulvotomentosa* Hsu et S.C.Cheng (131);
**121**	methyl 3-(3,4-dihydroxyphenyl)-propanoate	C_10_H_12_O_4_	*Eucommia ulmoides* Oliv (57).
**122**	methyl (2R,3S)-2,3-dihy-droxy-3-(4-methoxyphenyl)propanoate	C_11_H_14_O_5_	*Kaempferia galanga* L (136).
**123**	ethyl(2R,3S)-2,3-dihydroxy-3-(4-methoxyphenyl)propanoate	C_12_H_16_O_5_	*Kaempferia galanga* L (136).
**124**	ethyl rosmarinate	C_20_H_20_O_8_	*Prunella vulgaris* L (87).
**125**	ethyl caffeate	C_11_H_12_O_4_	*Lonicera fulvotomentosa* Hsu et S.C.Cheng (137); *Prunella vulgaris* L (113).
**126**	dihydroconiferyldihydro-p-coumarate	C_19_H_22_O_5_	*Dendrobium officinale* Kimura et Migo (138);
**127**	caftaric acid monomethyl ester	C_14_H_14_O_9_	*Cornus officinalis* Sieb. et Zucc (56)
**128**	cis ethyl p-methoxycinnamate	C_12_H_14_O_3_	*Kaempferia galanga* L (40).
**129**	angeliferulate	C_21_H_24_O_8_	*Angelica sinensis* (Oliv.) Diels (135).
**130**	butyl rosmarinate	C_22_H_24_O_8_	*Prunella vulgaris* L (87).
**131**	3,4,α-trihydroxy-methyl phenylpropionate	C_10_H_12_O_5_	*Prunella vulgaris* L (87).
**132**	2-{[3-(3,4- dihydroxyphenyl)propanoyl]oxy} propanoic acid methyl	C_13_H_16_O_6_	*Eucommia ulmoides* Oliv (57).
**133**	3,4,α-trihydroxy-butyl phenylpropionate	C_13_H_18_O_5_	*Prunella vulgaris* L (87).
**134**	(±)3-{2-[1-(3′,4′-dihydroxy-phenyl)ethyl]-4,5-dihydroxyphenyl} propanoic acid methyl	C_18_H_20_O_6_	*Eucommia ulmoides* Oliv (57).
**135**	(Z)-methyl p-hydroxycinnamate	C_10_H_10_O_3_	*Cannabis sativa* L (109).
**136**	caffeoyltartaric acid dimethyl ester	C_15_H_16_O_9_	*Cornus officinalis* Sieb. et Zucc (79)
Hydroxycinnate glycosides			
**137**	caffeoylglucose	C_15_H_18_O_9_	*Sterculia lychnophora* Hance (33); *Morus alba* L. (fruit) (82)
**138**	1-O-[(E)-p-Coumaroyl]-D-glucose	C_15_H_18_O_8_	*Alpinia oxyphylla* Miq (91).
**139**	sinapic acid glucoside	C_17_H_22_O_10_	Alpinia oxyphylla Miq (91); *Nelumbo nucifera* Gaertn. (fruit) (139)
**140**	3’-dehydroxyl-rosmarinic acid-3-o-*β*-D-glucoside	C_23_H_24_O_12_	*Perilla frutescens* (L.) Britt. (fruit) (64)
**141**	6-O-feruloyl-D-glucose	C_16_H_20_O_9_	*Alpinia oxyphylla* Miq (91).
**142**	caffeic acid 4-O-glucoside	C_15_H_18_O_9_	*Chrysanthemum morifolium* Ramat (115).
**143**	caffeic acid dihexoside	C_21_H_28_O_14_	*Codonopsis pilosula* (Franch.) Nannf (66).
**144**	caffeic acid trihexoside	C_27_H_38_O_19_	*Codonopsis pilosula* (Franch.) Nannf (66).
**145**	caffeic acid-3-O-glucoside	C_15_H_18_O_9_	*Phyllanthus emblica* L (71); *Piper nigrum* L (43); *Perilla frutescens* (L.) Britt. (fruit) (64)
**146**	codonosides A	C_38_H_48_O_20_	*Codonopsis tangshen* Oliv (140).
**147**	codonosides B	C_38_H_48_O_20_	Codonopsis tangshen Oliv (140).
**148**	coumaroylglucose	C_15_H_18_O_8_	*Perilla frutescens* (L.) Britt. (leaf) (130)
**149**	dihydroferulic acid hexoside	C_16_H_22_O_9_	*Codonopsis pilosula* (Franch.) Nannf (66).
**150**	ferulic acid 4-O-glucoside	C_16_H_20_O_9_	*Morus alba* L. (fruit) (82); *Sesamum indicum* L (18); *Piper nigrum* L (43).
**151**	regaloside B	C_20_H_26_O_11_	*Lilium lancifolium* Thunb (141).
**152**	regaloside C	C_18_H_24_O_11_	*Lilium lancifolium* Thunb (141).
**153**	regaloside E	C_18_H_24_O_10_	*Lilium lancifolium* Thunb (141).
**154**	rosmarinic acid glucuronide	C_24_H_26_O_13_	*Prunella vulgaris* L (113).
**155**	salviaflaside	C_24_H_26_O_13_	*Perilla frutescens* (L.) Britt. (leaf) (103); *Perilla frutescens* (L.) Britt. (fruit) (64); *Prunella vulgaris* L (142).
**156**	sinapate 4-O-*β*-D-glucopyranoside	C_17_H_22_O_10_	*Nelumbo nucifera* Gaertn. (fruit) (35)
**157**	dihydroferulic glucuronide	C_16_H_20_O_10_	*Prunella vulgaris* L (113).
Hydroxycinnate salts			
**158**	caffeic acid 3-sulfate	C_9_H_8_O_7_S	*Piper nigrum* L (43).

### 2.3 Hydroxyphenylacetic acids and other acids

**Figure 3:**
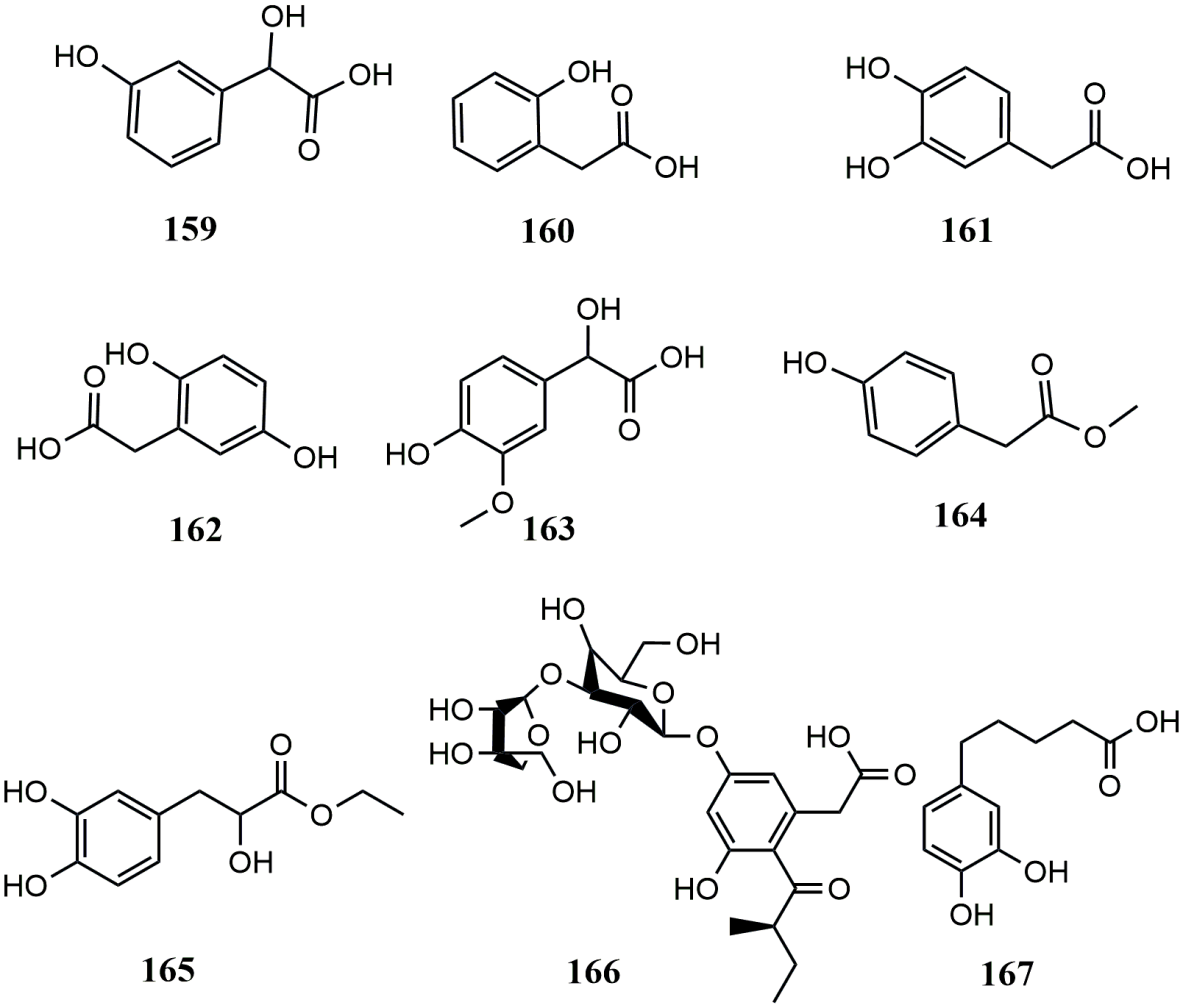
Structures of hydroxyphenylacetic acids from medicinal and edible homologous plants.

In contrast, hydroxyphenylacetic acids are the least abundant phenolic acids. Only 8 hydroxyphenylacetic acids have been reported in MEHPs, including 5 simple hydroxyphenylacetic acids, 2 hydroxyphenylacetates, and 1 hydroxyphenylacetate glycoside. There are only 10 MEHPs reported. In addition, another type of phenolic acid was found in Piper nigrum L.: 5-(3’,4’-dihydroxyphenyl)-valeric acid. Details are shown in **Table 3** and the structure is shown in **Figure 3**.

**Table 3:** Hydroxyphenylacetic acids in medicinal and edible homologous plants.

No.	Components	Molecular Formula	MEHPs
Simple hydroxyphenylacetic acids			
**159**	m-hydroxymandelic acid	C_8_H_8_O_4_	*Panax quinquefolium* L (27).
**160**	O-hydroxybenzene acetic acid	C_8_H_8_O_3_	*Hippophae rhamnoides* L (98).
**161**	3,4-dihydroxyphenylacetic acid	C_8_H_8_O_4_	*Sesamum indicum* L (18); *Piper nigrum* L (43).
**162**	homogentisic acid	C_8_H_8_O_4_	*Perilla frutescens* (L.) Britt. (leaf) (63)
**163**	homovanillic acid	C_9_H_10_O_4_	*Lycium barbarum* L (17); *Mentha haplocalyx* Briq (105).
Hydroxyphenylacetates			
**164**	4-hydroxyphenylacetic acid methyl ester	C_9_H_10_O_3_	*Morus alba* L. (fruit) (85)
**165**	ethyl 3,4-dihydroxy-phenyl lactate	C_11_H_14_O_5_	*Prunella vulgaris* L (87).
Hydroxyphenylacetate glycosides			
**166**	5,7-dihydroxy-4-((2R)-2-methylbutan-1-onyl)-phenylacetic acid 7-O-b-D-apiofuranosyl (1–3)-β-D-glucopyranoside	C_25_H_33_O_14_	*Pogostemon cablin* (Blanco) Benth (128).
Others			
**167**	5-(3’,4’-dihydroxyphenyl)-valeric acid	C_11_H_14_O_4_	*Piper nigrum* L (43).

We found that 5 MEHPs contain more than 20 phenolic acids: *Morus alba* L. (fruit) (26), *Piper nigrum* L (23), *Prunella vulgaris* L (23), *Sesamum indicum* L (22), *Perilla frutescens* (L.) Britt. (leaf) (20). Among these, *Prunella vulgaris* L. and *Perilla frutescens* (L.) Britt (leaf) belong to Lamiaceae, indicating that phenolic acids may be the main active compounds in Lamiaceae plants. Among the 167 identified MEHP phenolic acids, hydroxycinnamic acids were the most numerous and widely distributed, with chlorogenic acid present in 43 MEHPs, highlighting its accessibility and potential for development.

## 3 Anti-inflammatory activity and mechanism of MEHP phenolic acids

Recognizing the pivotal role of inflammatory response in inflammatory diseases, anti-inflammatory drugs occupy a central position in their management and treatment. The intricate relationship between the anti-inflammatory mechanism and inflammatory diseases underscores their interconnectedness. Presently, the anti-inflammatory drugs available in the market primarily function through various pathways, including nuclear factor- kappa B (NF-κB), mitogen activated protein kinase (MAPK), NOD-like receptor protein 3(NLRP3), nuclear factor E2-related factor 2 (Nrf2), toll-like receptors (TLRs), and interleukin-17 (IL-17). Additionally, the regulation of gut microbiota and immune response mechanisms contribute significantly to their effectiveness. Notably, MEHPs phenolic acids exhibit remarkable anti-inflammatory activity, as evidenced in numerous studies on inflammatory diseases. Their diverse anti-inflammatory mechanisms of action offer promising potential for further development.

### 3.1 NF-κB pathway

**Figure 4:**
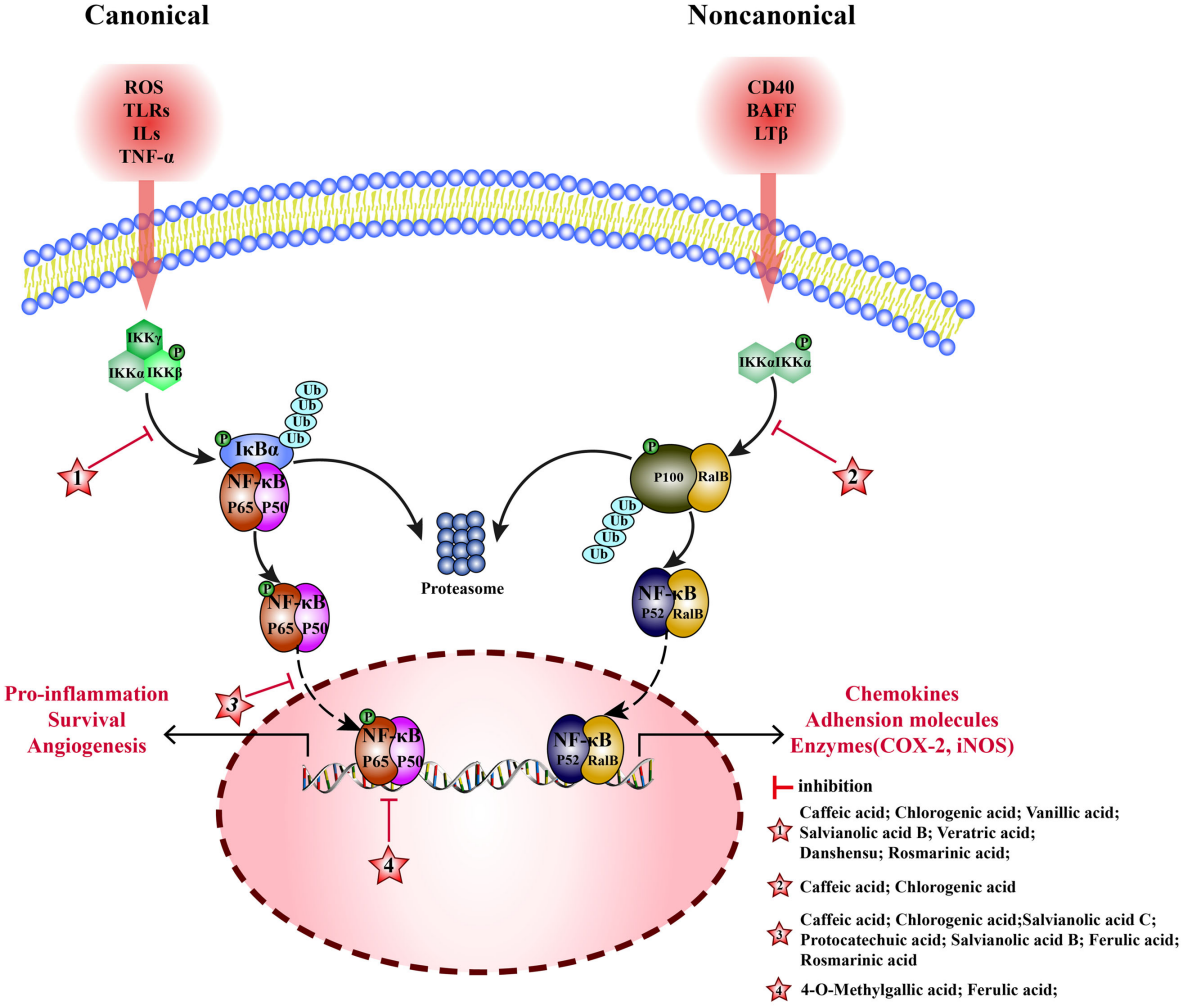
Molecular mechanism of the anti-inflammatory activity of phenolic acids from medicinal and edible homologous plants against the NF-κB pathway.

Nuclear factor- kappa B (NF-κB) is an important nuclear transcription factor in cells, formed by dimerization of Rel proteins (p50, p52, p65, c-Ral, and RalB). NF-κB pathway consists of canonical and non-canonical pathways. (1) Canonical: NF-κB (p65/p50) and inhibitor of NF-κB (IκBα) are bound in the cytoplasm with an inactive dimer. When subjected to reactive-oxygen species (ROS), toll-like receptors (TLRs), interleukin 1β (IL-1β) and tumor necrosis factor α (TNF-α), inhibitor of κB kinase (IKKβ) is activated, then IκB is degraded, and p65/p50 dimer is dissociated. Subsequently, p65 is phosphorylated and translocated to the nucleus to activate the target genes, inducing the transcription of TNF-α, IL-1β, and interleukin 6 (IL-6) (143). (2) Noncanonical: RalB binds to p100 as inactive dimer in the cytoplasm. Lymphotoxin β(LTβ), B cell activating factor (BAFF), and tumor necrosis factor receptor superfamily member 5(CD40) stimulate the accumulation of NF-κB-inducing kinase (NIK) and activate IKKα. Then, p100 is degraded to p52, and the RalB/p52 dimer is translocated to the nucleus, to induce the transcription of related genes (144). MEHP phenolic acids can inhibit the NF-κB pathway by inhibiting canonical and non-canonical pathways (**Figure 4**).

#### 3.1.1 Inhibiting the canonical pathway

In canonical pathway, MEHP phenolic acid can inhibit the NF-kB pathway by suppressing the activation and nuclear translocation of NF-kB and blocking the binding of NF-kB to target genes.

Various hydroxycinnamic acids with caffeic acid as the parent nucleus, such as caffeic acid, chlorogenic acid, salvianolic acid B, and rosmarinic acid, have inhibitory effects on multiple links of canonical NF-kB pathway. Caffeic acid inhibits phosphorylation of IKKα/β and IκBα to inhibit activation of NF-κB, playing a key role in anti-rheumatoid arthritis (145), it can also inhibit nuclear translocation of p-p65, and alleviating inflammation to protect ischemia/reperfution(I/R)-injury in rats (25). Chlorogenic acid inhibits phosphorylation of IκBα and the p65 protein levels to interfere with NF-κB pathway, showing anti-arthritis (146) and anti-mastitis (147) effects. Meanwhile, it inhibits nuclear translocation of p-p65 to block NF-κB signaling pathway alleviating LPS-induced inflammation in Caco-2 (148), RAW 264.7 (149), and rat hepatic stellate cells (150). salvianolic acid B inhibits activation of NF-κB pathway by inhibiting the phosphorylation of p65 (151), alleviating inflammation of arthritis mouse, it can also reduce the release of TNF-α, IL-1β, and IL-6 by inhibiting nuclear translocation of p65 producing anti-atherosclerotic effect (152). In an inflammatory model of human skin fibroblasts (HSF) induced by TNF-α, rosmarinic acid has been shown to inhibit the phosphorylation and degradation of IκBα and the activation of NF-κB (153). Additionally, rosmarinic acid can alleviate acute pancreatitis induced by sodium taurocholate by inhibiting the nuclear translocation of p65 (154). In LPS-induced acute kidney injury in mice, ferulic acid can inhibit inflammation by inhibiting nuclear translocation of p65 (155). Moreover, ferulic acid cuts off the combination between p-NF-κB and the transcription factor cAMP-response element binding protein(CREB), inhibiting NF-κB binding to DNA, helping repair acute liver injury induced by cecal ligation perforation (CLP) in mice (156).

Furthermore, two additional hydroxycinnamic acids have also been reported to have inhibitory effects on the canonical NF-kB pathway. Danshensu reduces expression of p-IKKα/β, p-IκBα, and p-p65, and upregulates expression of IκBα to alleviate chronic kidney disease in mice (157). Salvianolic acid C blocks NF-κB signaling pathway by inhibiting nuclear translocation of p-p65 and suppresses inflammation in BV2 cells induced by LPS (158).

Four hydroxybenzoic acids also possess inhibitory effects on the canonical NF-kB pathway. Vanillic acid inhibits phosphorylation IκBα, alleviating inflammation of chondrocytes in patients with arthritis (159). In acute lung injury mice, veratric acid inhibits the phosphorylation of IκB and p65, regulating the NF-κB signaling pathway to alleviate inflammatory damage induced by LPS (160). Protocatechuic acid inhibits nuclear translocation of p65, protein and mRNA expression of TNF- α, IL-1 β, and IL-6 in SH-SY5Y cells, and promotes repair after cerebral hemorrhage in mice (161). 4-O-methylgallic acid can modify the DNA binding domain of NF-κB to directly block NF-κB binding with DNA in the nucleus, thereby inhibiting leukocyte adhesion to endothelial cells and preventing vascular inflammation (162).

#### 3.1.2 Inhibiting the noncanonical pathway

In noncanonical pathways, an accumulation of NIK promotes phosphorylation of IKKα, activating NF-κB (RalB/p52); therefore, NIK is a key kinase. Two hydroxycinnamic acids belonging to the caffeic acid category have been found to exert inhibitory effects on the noncanonical NF-kB pathway. Caffeic acid inhibits phosphorylation of NIK and IKK and the activation of noncanonical NF-κB pathway, alleviating inflammation in endothelial cells (163). Chlorogenic acid inhibits the expression of RalB and p52 to exert anti- liver cancer effects (164).

In comparison to non-canonical pathways, MEHP phenolic acids exert a more pronounced inhibitory effect on canonical pathways. Seven hydroxycinnamic acids and four hydroxybenzoic acids possess inhibitory effects on the non-pharmacological pathway, demonstrated across various models and conditions. Notably, four caffeic acid-like hydroxycinnamic acids are capable of simultaneously targeting diverse stages of the canonical pathway to alleviate inflammatory conditions, including NF-kB activation, nuclear translocation, and binding to target genes. Furthermore, two types of hydroxycinnamic acid, both belonging to the caffeic acid category, can inhibit the non-pharmacological pathway.

### 3.2 MAPK pathway

**Figure 5:**
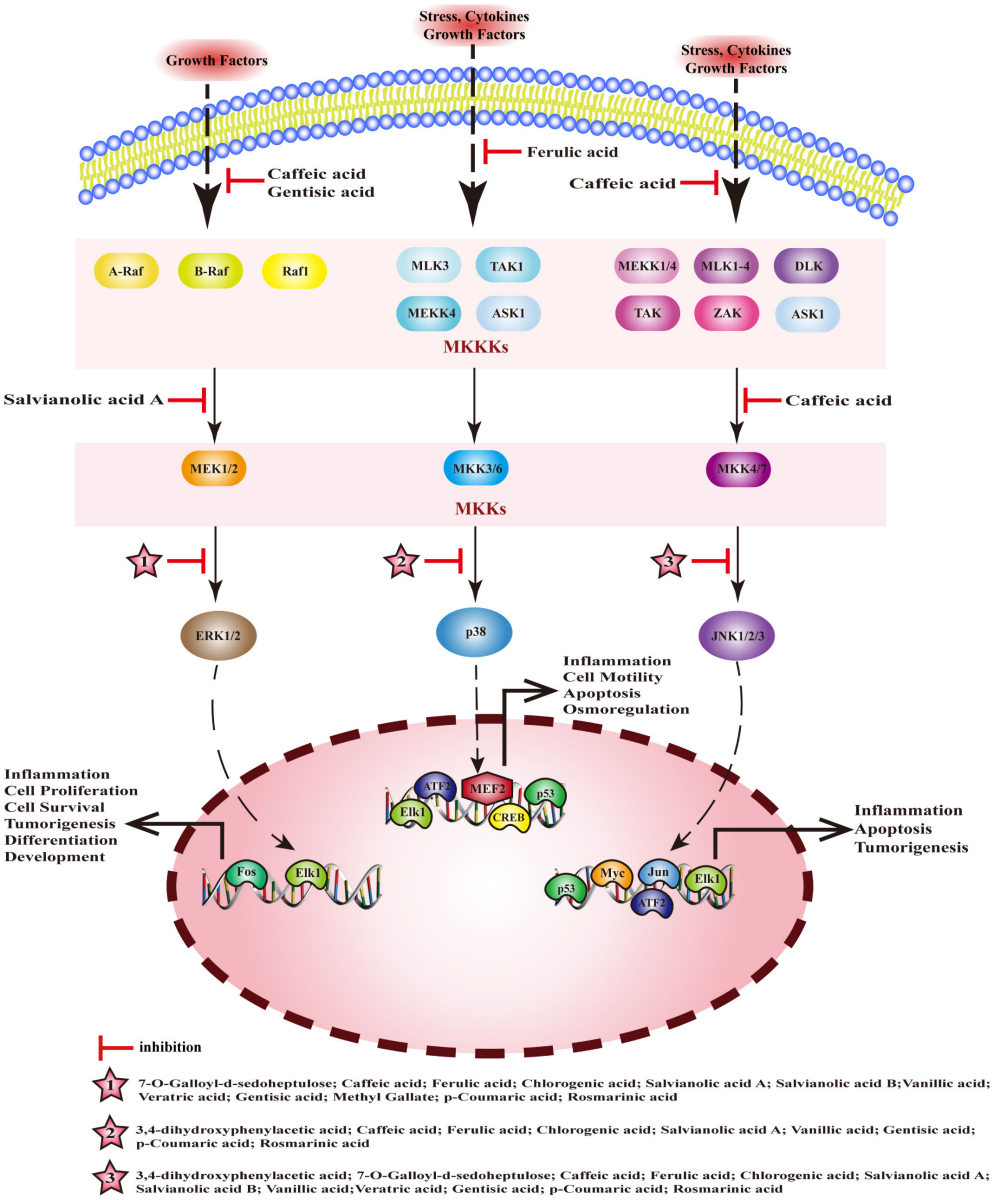
Molecular mechanism of the anti-inflammatory activity of phenolic acids from medicinal and edible homologous plants against the MAPK pathway.

Mitogen activated protein kinase (MAPK) is a serine threonine protein kinase. The MAPK pathway is composed of a tertiary kinase pattern, including mitogen-activated protein kinase kinase kinase (MKKKs), mitogen-activated protein kinase kinases (MKKs), and MAPKs. MAPKs comprises four subfamilies: extracellular regulated protein kinases (ERK), mitogen-activated protein kinase p38 (p38), c-Jun N-terminal kinase (JNK), and extracellular regulated protein kinases 5 (ERK5), and these pathways are named accordingly. Among them, ERK, p38, and JNK are the three canonical MAPK pathways, which are closely associated with inflammation. MEHP phenolic acids can inhibit the MAPK pathway and exert anti-inflammatory activity by inhibiting the activation of kinases (**Figure 5**).

#### 3.2.1 Inhibiting MKKKs

The MKKKs family of Serine/threonine-protein kinase (Raf), encompassing A-Raf, B-Raf, and Raf1, plays a pivotal role in the activation of the ERK pathway. Concurrently, MKKKs such as serine/threonine-protein kinase RIM15 (TAK1), mitogen-activated protein kinase kinase kinase (MEKK), and mitogen-activated protein kinase kinase kinase 5 (ASK1) are instrumental in triggering the p38 and JNK pathways. Gentisic acid inhibits the expression of Raf in ankle and knee tissues and regulates Raf/ERK signaling, thus alleviating rheumatoid arthritis in rats (165). Caffeic acid exerts anti-gastritis effects by inhibiting interleukin-1 receptor-associated kinase 1 (IRAK1), interleukin-1 receptor-associated kinase 4 (IRAK4), and TAK1 by interfering with the JNK/MAPK pathway (166). Caffeic acid can inhibit the phosphorylation of c-Raf and the activation of ERK1/2, reduce the release of inflammatory factors, and exert a detoxifying effect on liver toxicity induced by acetaminophen(APAP) in mice (167). Ferulic acid alleviates LPS-induced inflammation of RAW 264.7 cells by inhibiting the phosphorylation of TAK1, interfering with the p38/MAPK pathway to inhibit the activation of NF-κB (168).

#### 3.2.2 Inhibiting MKKs

The three canonical MAPK pathways correspond to distinct MKKs, the ERK pathway is associated with MEK, the p38 pathway aligns with MKK3/6, and the JNK pathway is linked to MKK4/7. Two hydroxycinnamic acids, with caffeic acid serving as their central component, exert a significant inhibitory influence on MKKs. Caffeic acid can exert anti-gastritis effects by inhibiting MKK4/7 to inhibit the JNK/MAPK pathway (166). Salvianolic acid A can effectively mitigate the inflammatory response in the lungs of patients suffering from acute lung injury by suppressing LPS-induced phosphorylation of MEK, and ERK within the lung tissue (169).

#### 3.2.3 Inhibiting MAPKs

In mice with colitis, chlorogenic acid reduces the expression ERK1/2, p-ERK, p38, p-p38, JNK, p-JNK, p-IκB, and p-p65 in tissues, blocks the ERK/JNK pathway, and reduces symptoms of colitis (170). In rats with arthritis, p-coumaric acid promotes the inactivation of MAPK pathway, inhibits inflammation, cartilage degeneration, and osteoclast formation by downregulating the expression of JNK, p-JNK, and ERK1/2[187]; it can also inhibit the expression of p-p38/pJNK/pERK and p-IKKβ/p-IκB/NF-κB, block caspase-1/MAPK/NF-κB signaling cascade to inhibit the inflammation of activated mast cell and splenocyte[188].Ferulic acid can inhibit NF-κB pathway by reducing the phosphorylation of p38 and JNK, thereby preventing endometritis (171). Salvianolic acid A inhibits the activation of p38, JNK, and ERK, blocking the activation of MAPK pathways, and exerts anti-inflammatory effects in mice with arthritis (172). 3,4-dihydroxyphenylacetic acid inhibits inflammation and repairs the intestinal barrier dysfunction in mice with type 2 diabetes by inhibiting the activation of JNK and p38 (173). 7-O-galloyl-d-sedoheptulose can inhibit the activation of NF-κB and AP-1 and plays a key role in liver protection in type 2 diabetes by inhibiting phosphorylation of ERK1/2 and JNK (174). Caffeic acid significantly inhibits the expression of p-p38, regulates inflammation and apoptosis through p53 and p38/MAPK signaling pathways, and prevents atherosclerosis (175). In addition, it can inhibit the phosphorylation of JNK, p38, and c-Jun in a dose-dependent manner, and block phosphorylation of ERK1/2 to alleviate LPS-induced inflammation in bovine mammary epithelial cells (bMECs) (176). Salvianolic acid B can downregulate the expression of p-ERK and p-JNK, inhibit the transcription of inflammatory factors, and produce anti-pneumonia effects (177). Vanillic acid significantly reduces the levels of pERK, pJNK, and p-p38, regulates the NF-κB/MAPKs signaling pathway to alleviate the allergic inflammation of HMC-1 (178). Methyl gatellate can inhibit LPS-induced inflammation in mouse macrophages by inhibiting the activation of ERK (179). Rosmarinic acid can inhibit the activation of ERK, JNK, and p38, block MAPK/NF-κB signaling pathway to improve LPS-induced inflammation in vascular smooth muscle cells (180).

When it comes to inhibiting the MAPK pathway, MEHP phenolic acids demonstrate the most profound inhibitory effect on MAPKs. Among the compounds tested, seven hydroxycinnamic acids, three hydroxybenzoic acids, and one hydroxyphenylacetic acid all exhibit inhibitory effects on MAPKs, with most of them capable of suppressing multiple types of MAPKs. In terms of inhibiting MKKKs, one hydroxybenzoic acid and two hydroxycinnamic acids are effective, while two caffeic acid-like hydroxycinnamic acids specifically demonstrate an inhibitory effect on MKKs.

### 3.3 NLRP3 pathway

**Figure 6:**
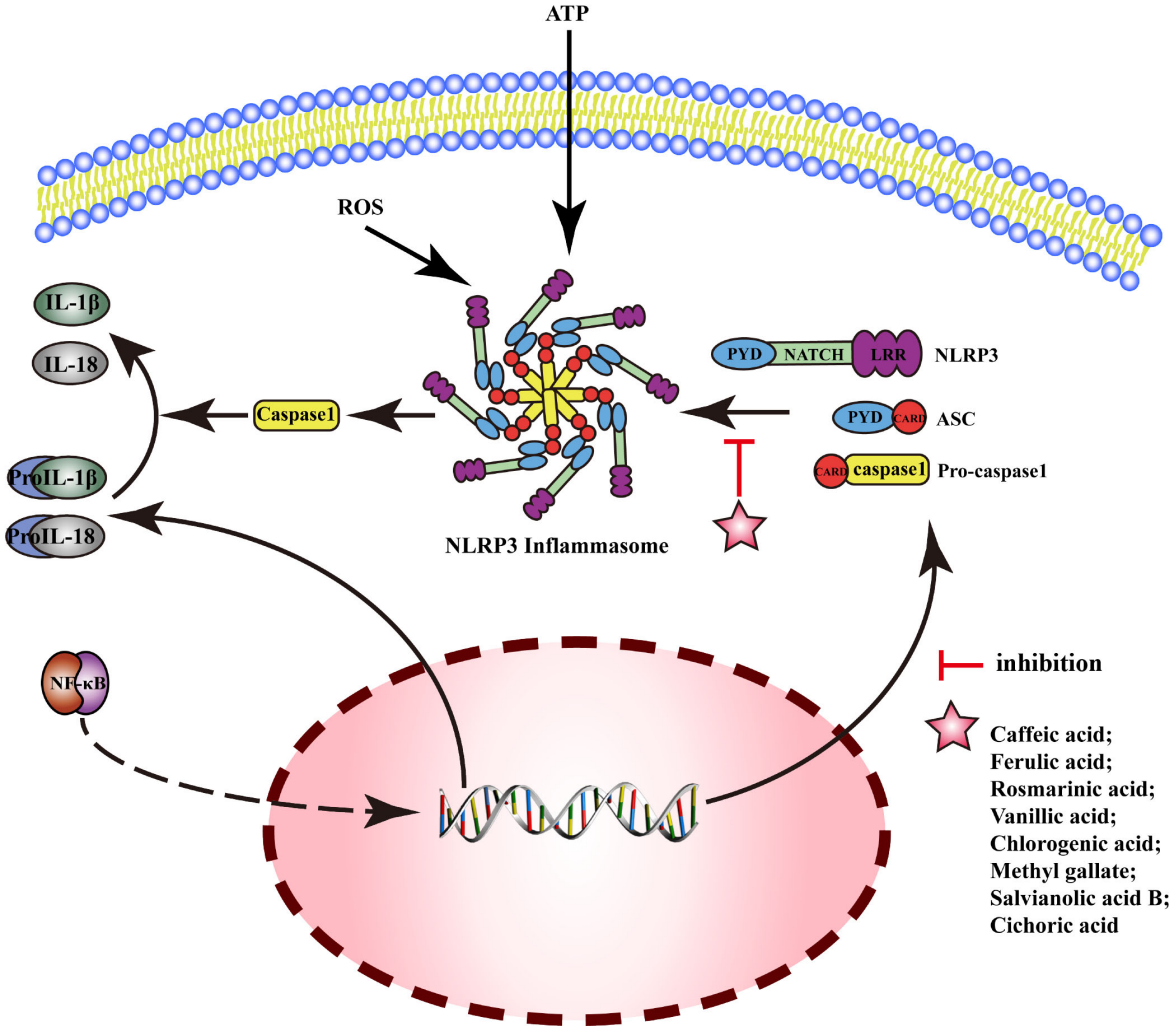
Molecular mechanism of the anti-inflammatory activity of phenolic acids from medicinal and edible homologous plants against the NLRP3 pathway.

NOD-like receptor protein 3(NLRP3) is an inflammasome sensor protein, and the activation of NLRP3 can generate an oligomer complex “Inflammamasone”, which includes apoptosis-associated speck-like protein containing CARD(ASC) and caspase-1. The activation of the typical NLRP3 inflammasome pathway requires two stages: Signal 1 (priming): upregulation of the protein expressions related to inflammasomes (including inflammasome sensor proteins, IL-1β, and IL-18) by upregulating the transcriptional activity of NF-κB. Signal 2 (activation): NLRP3 interacts with pro-caspase-1 after assembly with ASC, then produces a large amount of caspase-1, which catalyzes the dissociation of pro-IL-1β and pro-IL-18 and initiates inflammatory response. MEHP phenolic acids can exert anti-inflammatory activity by inhibiting the NLRP3 pathway; the mechanism is shown in **Figure 6**.

In acute gouty arthritis, ferulic acid can exert anti-inflammatory effects by inhibiting the activation of NLRP3 inflammasomes (181). Caffeic acid downregulates mRNA expression of IL-1β and IL-18 to reduce inflammatory reaction of human umbilical vein endothelial cell (HUVEC) induced by advanced glycation end products (AGEs) (182). Rosmarinic acid exerts anti-inflammatory effects by inhibiting the activation and assembly of NLRP3 inflammasomes in psoriasis (183), liver injury (184), and neuroinflammation (185). Vanillic acid can inhibit the activation of NLRP3 inflammasomes and the expression of IL-18 and IL-1β to alleviate arthritis in rats by downregulating the expression of caspase-1, ASC, and NLRP3 (186). Chlorogenic acid improves pneumonia induced by Klebsiella pneumoniae (187), and inhibits periodontal disease (188) by inhibiting activation of NLRP3 inflammasome. Methyl gallate can inhibit the assembly of NLRP3 inflammasome by blocking oligomerization of NLRP3 to alleviate the inflammatory response in mice with hyperuricemic nephropathy (189). Salvianolic acid B attenuates cell death mediated by endoplasmic reticulum stress, by inhibiting NLRP3 inflammasome and reducing the secretion of caspase-1, IL-1β, and IL-18 (190). Cichoric acid decreases the levels of NLRP3, IL-1β, caspase-1, ASC oligomer, and ASC monomer and the release of IL-1β and TNF-α, inhibiting the inflammation in THP-1-derived macrophages (THP-Ms) induced by monosodium urate (MSU) (191).

Reports indicate that two hydroxybenzoic acids and six hydroxycinnamic acids possess the ability to suppress the NLRP3 pathway. Notably, five of these hydroxycinnamic acids share caffeic acid as their common backbone, suggesting that caffeic acid-derived phenolic acids exert the most pronounced inhibitory effect on the NLRP3 pathway.

### 3.4 Nrf2 pathway

**Figure 7:**
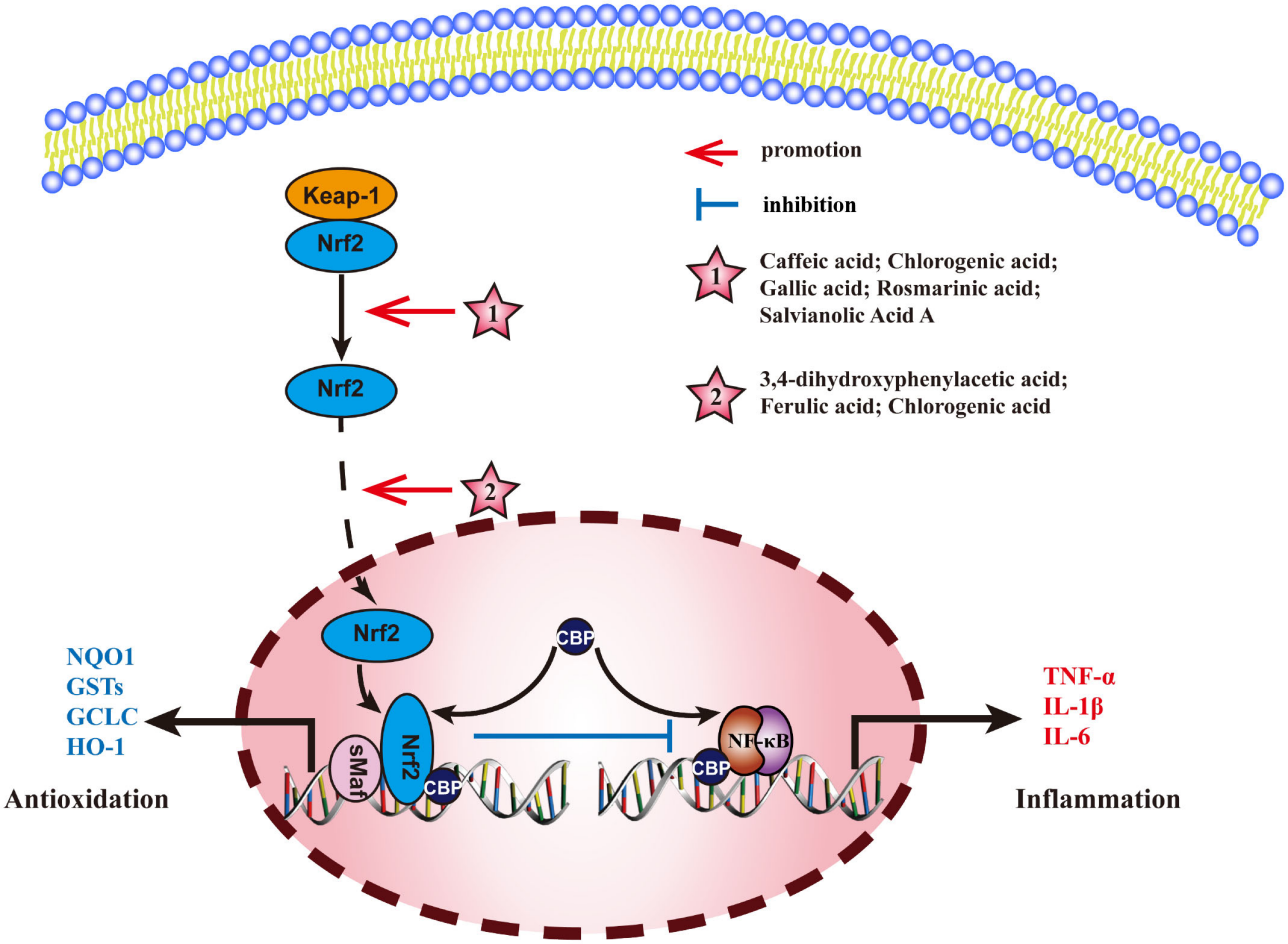
Molecular mechanism of the anti-inflammatory activity of phenolic acids from medicinal and edible homologous plants against the Nrf2 pathway.

Nuclear factor E2-related factor 2 (Nrf2) is a key transcription factor, that normally, binds to kelch-like ECH-associated protein 1(Keap1) in the cytoplasm, rapidly degrading under the action of ubiquitin proteasome pathway. When cells are stimulated by ROS or other nucleophilic agents, Nrf2 uncouples with Keap1 and is activated by phosphorylation. It is then transported into the nucleus where it competes with p65/p50 to activate the transcription factor CBP, inhibits the binding of p65/p50 to target genes and reduce the transcription of TNF-α, IL-1β, and IL-6 to inhibit the inflammatory response. Therefore, activation of Nrf2 and nuclear translocation of Nrf2 are key links in regulating Nrf2 pathway. The mechanism of MEHP phenolic acids exerting anti-inflammatory activity through Nrf2 pathway is shown in **Figure 7**.

#### 3.4.1 Promoting the activation of Nrf2

Caffeic acid exerts an anti-hepatitis effect by upregulating the expression and phosphorylation of P62 (an autophagy substrate), promoting its binding and degradation with Keap1, inducing an increase in Nrf2 expression (192). Chlorogenic acid activates Nrf2/HO-1 pathway to alleviate oxidative stress and inflammatory response, repairs intestinal barrier, and effectively improves DSS-induced colitis (193). Gallic acid inhibits NF-κB pathway by binding to Keap1 and mediating Nrf2 activation, thus exerting anti-pneumonia effect (194). Rosmarinic acid can bind to Keap1, blocking the association between Keap1 and Nrf2 and activating Nrf2, thereby relieving bacterial pneumonia (195). Salvianolic acid A can directly bind to Keap1, promote the activation of Nrf2, and alleviate the inflammatory response in Schwann cells induced by high glucose (196).

#### 3.4.2 Promoting nuclear translocation of Nrf2

3,4-dihydroxyphenylacetic acid can inhibit ethanol-induced hepatotoxicity by increasing Nrf2 protein expression and nuclear translocation (197). Ferulic acid increases the nuclear translocation of Nrf2 to inhibit LPS-induced inflammation in bMECs (198). Chlorogenic acid can improve ischemic brain injury (199), relieve endometritis (200), and regulate blood sugar (201) by increasing nuclear translocation of Nrf2 and inhibiting NF-κB pathway.

Among all the MEHP phenolic acids, hydroxycinnamic acid stands out for its remarkable promoting effect on the Nrf2 pathway. Specifically, five hydroxycinnamic acids, all belonging to the caffeic acid family, can enhance the activation of Nrf2. Furthermore, gallic acid, a hydroxybenzoic acid, also demonstrates a similar effect. Additionally, two hydroxycinnamic acids and one hydroxyphenylacetic acid contribute to the nuclear translocation of Nrf2. Notably, chlorogenic acid is unique in its ability to concurrently promote both the activation and nuclear translocation of Nrf2, thereby exerting significant anti-inflammatory effects.

### 3.5 TLRs pathway

**Figure 8:**
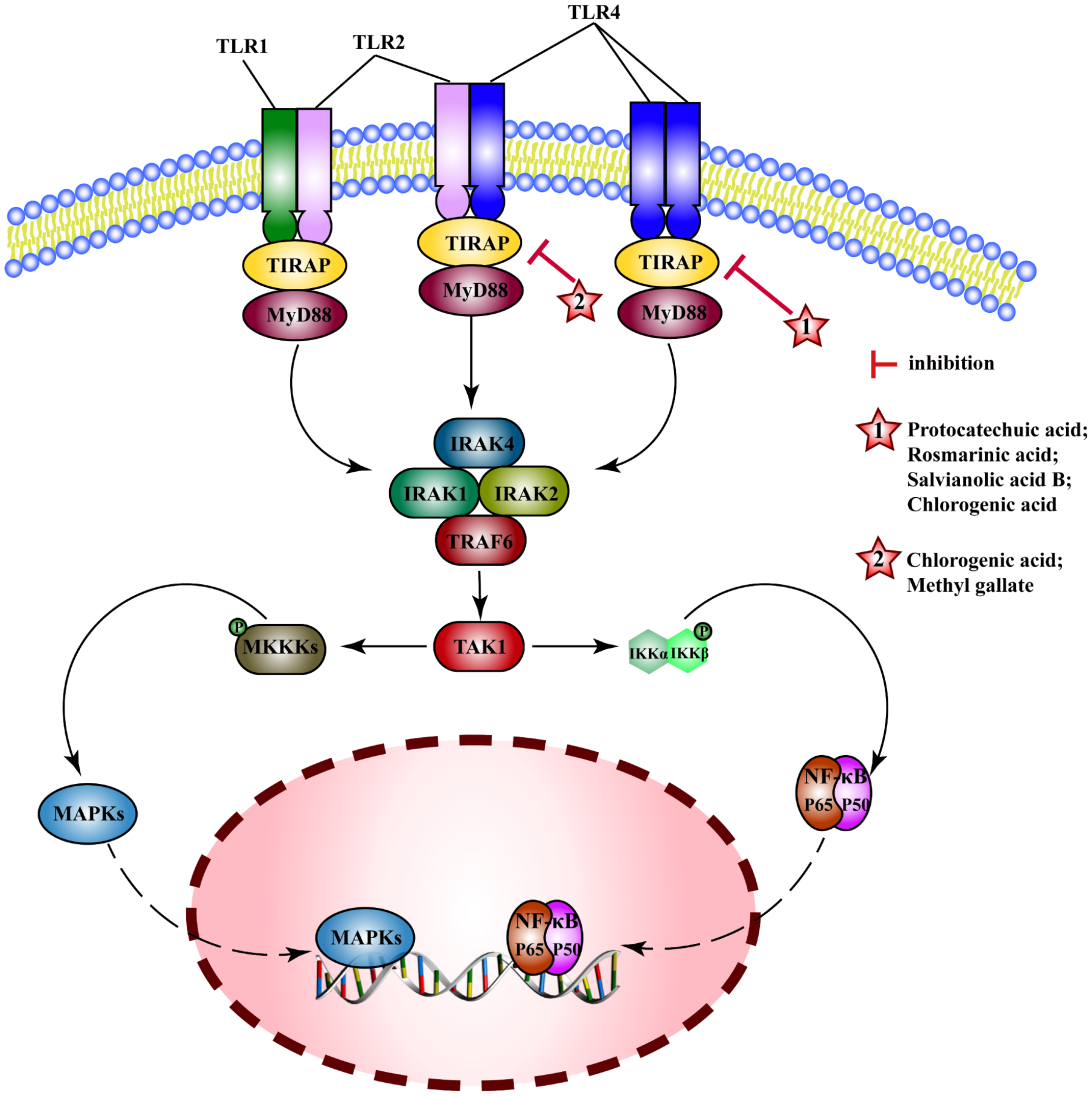
Molecular mechanism of the anti-inflammatory activity of phenolic acids from medicinal and edible homologous plants against the TLRs pathway.

Toll-like receptors (TLRs) are pattern recognition receptors (PRRs) that recognize microorganisms when they invade the body and activate immune responses. In general, TLRs mainly transduce signals through myeloid differentiation factor 88(MyD88) or TIR-domain containing adaptor inducing interferon-β(TRIF) pathways. MyD88 signals induce the production of inflammatory factors (such as TNF, IL-6, IL-1β) and chemokines (such as C-C motif ligand 4, CCL4). MyD88 binds to TLRs and recruits IRAK4 and IRAK1/2 to Myddosome, which activates (TNF receptor-associated factor 6) TRAF6, induces the activation of NF-κB and MAPK pathways and the expression of proinflammatory cytokines (202). MEHP phenolic acids exert anti-inflammatory effects mainly by interfering with the TLRs/MyD88 pathway (**Figure 8**).

For influenza A in mice (203), acute pancreatitis in rats (204), protocatechuic acid alleviates inflammatory response by reducing the activation of TLR4 and inhibiting NF-κB pathway. Rosmarinic acid can improve neuroinflammation after spinal cord injury (205), relieve hyperlipidemia (206), and inhibit mastitis (207) by inhibiting TLR4/MyD88-NF-κB pathway. Salvianolic acid B attenuates PM 2.5-induced tracheitis in mice by inhibiting TLR4, MyD88, and TRAF6, interfering MAPK pathway and blocking NRLP3 activation (208). Chlorogenic acid inhibits expression of TLR4 and MyD88, interferes with their downstream pathways to improve the intestinal barrier damage in weaned piglets (209), alleviates hepatitis in mice (210) and alcoholic hepatitis in rats (211), and reduces inflammation in mouse glial cells (BV2) (212) and human gingival fibroblasts (HGFs) (213) induced by LPS, and Escherichia coli-induced inflammation in sheep endometrial epithelial cells (SEECs) (214). Chlorogenic acid may also exert anti-inflammatory effects by interfering with other TLRs such as, by inhibiting TLR2/TLR9-Myd88 signaling pathway to attenuate the inflammatory response in herpes encephalitis (215), down-regulating expression of TLR2/4 to decrease activity of NF-κB signaling pathway in epidermal cells, and inhibiting skin inflammation in mice (216). Methyl gallate can inhibit the activation of TLR2 to inhibit NF-κB and MAPK pathway and alleviate toe swelling in mice (217).

Researchers have identified the anti-inflammatory potential of MEHP phenolic acids, primarily by modulating the TLR/MyD88 pathway, showcasing their efficacy in various inflammatory models. Two hydroxybenzoic acids and three caffeic acid based hydroxycinnamic acids exhibit inhibitory effects on the TLRs pathway. Specifically, protocatechuic acid, rosmarinic acid, and salvianolic acid B can suppress the TLR4 pathway, whereas methyl gallate demonstrates inhibitory action towards the TLR2 pathway. Remarkably, chlorogenic acid possesses the ability to simultaneously inhibit the TLR2, TLR4, and TLR9 pathways, thereby exerting anti-inflammatory effects in a diverse range of diseases. These findings signified a wide spectrum of potential MEHP phenolic acid-mediated therapeutic interventions targeting the TLR-mediated inflammatory pathways.

### 3.6 IL-17 pathway

Interleukin-17 (IL-17) is a potent pro-inflammatory cytokine, which binds to its receptor IL-17R and activates TRAF6 through Act1, leading to the triggering of NF-kB and MAPK pathways.

**Figure 9:**
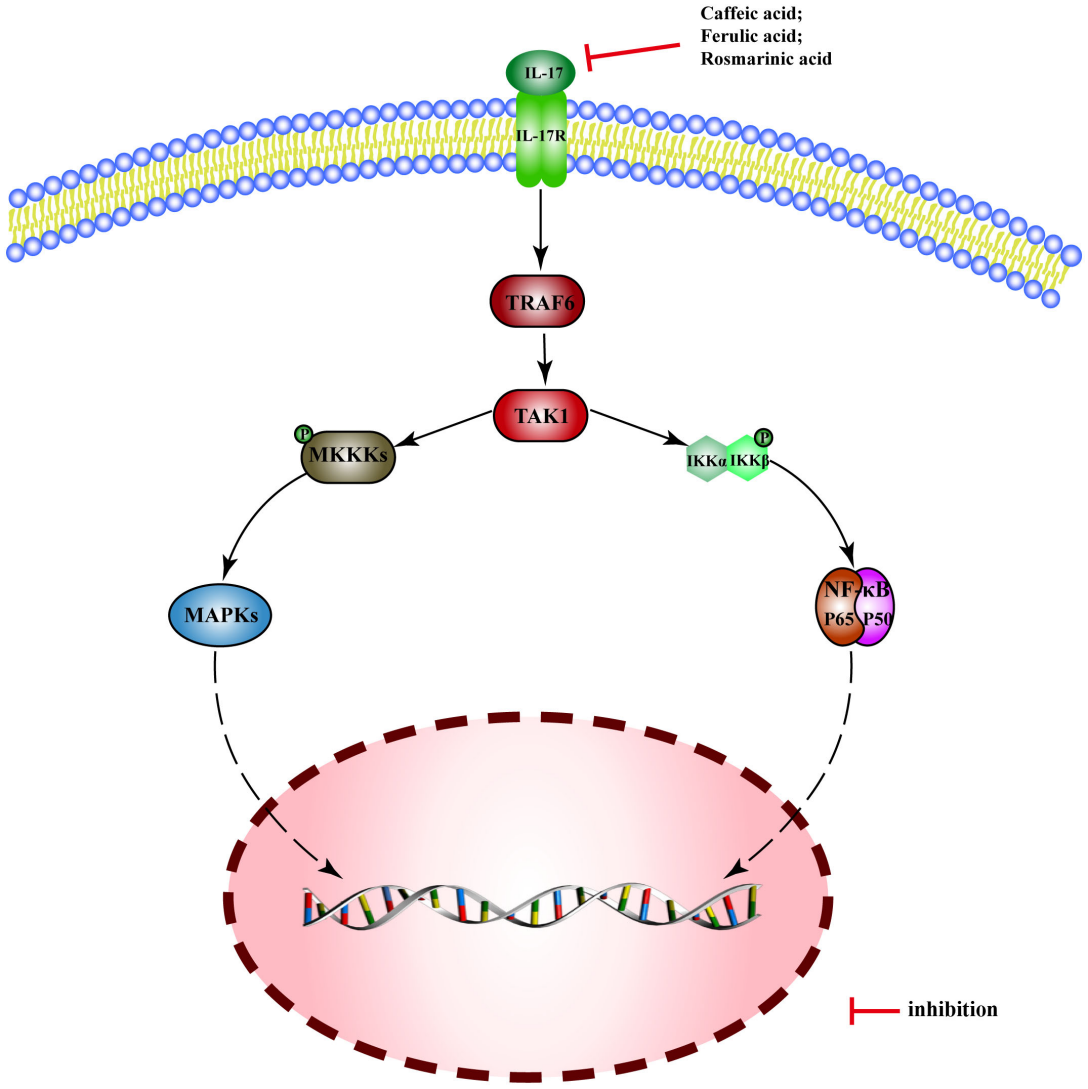
Molecular mechanism of the anti-inflammatory activity of phenolic acids from medicinal and edible homologous plants against the IL-17 pathway.

There are few reported MEHP phenolic acids that can regulate the IL-17 pathway, only three of which are hydroxycinnamic acid. Caffeic acid inhibits expression of IL-17 mRNA in intestinal tissue and alleviates DSS-induced colitis in mice (218). Ferulic acid inhibits secretion of IL-17 and blocks the combination of IL-17A and IL-17RA, thus improving skin inflammation in psoriatic mice (219). Rosmarinic acid can alleviate psoriasis-like dermatitis in mice by decreasing the differentiation of Th17 cells and inhibiting the expression of IL-17A (220) (**Figure 9**).

### 3.7 Regulating intestinal microflora

Numerous studies show that intestinal microbial species are closely related to anti-inflammatory effects and the by-products of bacterial metabolism, including some short-chain fatty acids (SCFA), can play a role in inhibiting inflammation. Ferulic acid significantly increases intestinal SCFA producing bacteria, as *Olsenella, Eisenbergiella, Dubosiella, Clostridiales_unclassified*, and *Faecalibaculum*, reduces endotoxin-producing and obesity-related bacteria, and inhibits the intestinal barrier functional damage induced by a high-fat diet in mice (221). Chlorogenic acid increases the abundance of SCFA-producing bacteria, such as *Dubosiella, Romboutsia, Mucispirillum*, and *Faecalibaculum*, as well as *Akkermansia*, enhanced the integrity of the intestinal barrier, while successfully preventing glucose metabolic disorders and endotoxemia (222).

Gut microbiota abundance and richness are closely associated with inflammation. The increase of Firmicutes and the decrease of Bacteroidetes could inhibit the development of inflammation (223). Protocatechuic acid can enhance the diversity of cecal microbiota, decrease the occurrence of Bacteroidota, Proteobacteria, and *Escherichia Shigella*, while promoting the abundance of Firmicutes and *Lactobacillus*, and mitigating *Salmonella Typhimurium*-induced intestinal barrier damage and inflammatory response in yellow chickens (224). Syringic acid enriches the abundance of *Alistipes* and *norank_f_norank_o_Gastranaerophilales* in mice, improving intestinal inflammation (225). Caffeic acid modulates the composition of the gut microbiome by reducing the relative abundance of *Bacteroides* and *Turicibacter*, while simultaneously increasing the relative abundance of *Alistipes* and *Dubosiella*, enhancing the abundance of *Dubosiella* and *Akkermansia*, effectively alleviating DSS-induced colitis in mice (226). Vanillic acid improves LPS-induced intestinal inflammation in weaned piglets by increasing the proportion of Firmicutes/Bacteroidetes, reducing the abundance of Prevotellaceae, and increasing the abundance of *Lachoiraceaea*, *Lachnospira*, *Eubacterium eligens*, and *Eubacterium (* 227). Chlorogenic acid can alleviate colitis induced by a high fat diet in obese rats by reducing the abundance of *Blautia*, *Sutterella*, and *Akkermansia* bacteria and increasing the abundance of *Ruminococcus (* 228).

The gut microbiota boasts a rich and diverse composition, and MEHP phenolic acids can exert anti-inflammatory effects by enhancing its diversity and modulating its richness. Notably, two hydroxybenzoic acids and four hydroxycinnamic acids possess significant effects, with ferulic acid and chlorogenic acid can increase the abundance of bacteria responsible for producing short-chain fatty acids (SCFAs), thereby promoting their production and exerting anti-inflammatory benefits.

### 3.8 Regulating immune responses

Immune response is a self-protective function of the body, where the appropriate immune response can clear pathogens, but excessive immune response can cause harm to the body; inflammation is a result of a severe immune response. Five distinct types of caffeic acid based hydroxycinnamic acid exhibit outstanding immune responses regulatory effects. Ethyl caffeate can alleviate collagen-induced arthritis in mice by inhibiting Th1 immune response and IFNγ-related signaling pathways (229). Salvianolic acid A regulates the immune response of dermis and inhibits the immune response of Th2/Th17/Th1 to alleviate atopic dermatitis in mice (230). Salvianolic acid B increases the percentage of CD3^+^CD4^+^/CD3^+^CD8^+^, restores balance of Th1 and Th2 type cytokines to inhibit the inflammatory response induced by a high fat diet (231). Rosmarinic acid inhibits production of IFN-γ and IL-4 from activated CD4^+^ cells, reduces the infiltration of CD4^+^, CD8^+^, and mast cells, slowing down the development of mouse atopic dermatitis (232). Chlorogenic acid can inhibit microglial polarization toward the M1 phenotype and improve neuroinflammation (233).

Collectively, these findings underscore the potent immune-regulatory capabilities of caffeic acid-based hydroxycinnamic acids in various inflammatory and autoimmune conditions, highlighting their potential for therapeutic applications in immune-mediated diseases.

## 4 Conclusion and future prospects

MEHP phenolic acids exhibit strong and varied anti-inflammatory mechanisms, highlighting their potential for therapeutic innovations. Their action in crucial pathways like NF-κB, MAPK, NLRP, Nrf2, TLRs, and IL-17, along with the regulation of gut microbiota and immune responses, amplifies their effectiveness.

Overall, hydroxycinnamic acid displays the most potent anti-inflammatory activity among MEHP phenolic acids, likely due to its carboxyl group’s adjacent double bond. The number, position, and types of substituents on hydroxyl groups significantly affect the anti-inflammatory effects. Compounds like protocatechuic acid, 4-O-methylgallic acid, 3,4-dihydroxyphenylacetic acid, gentic acid, gallic acid, danshensu, caffeic acid, etc with two or more hydroxyl groups, mainly have hydroxyl substitutions in the para position. Phenolic acid molecules with alkoxy (e.g., methoxy) or alkyl (e.g., methyl) substituents might enhance their compatibility with biomolecules (like enzymes or receptors) by increasing their lipid solubility or by stabilizing hydroxyl radicals, thus amplifying their anti-inflammatory potential. This is observed in compounds such as vanillic acid, ferulic acid, etc. Specifically, phenolic acids with catechol-like configurations, exhibiting two adjacent hydroxyl groups, are characterized by their robust antioxidant capabilities, enabling them to effectively neutralize free radicals and display pronounced anti-inflammatory properties. This is exemplified by Protocatechuic acid among all coffee acid derivatives. Caffeic acids, including caffeic acid, rosmarinic acid, chlorogenic acid, etc stand out due to their structural benefits, playing pivotal roles across varied anti-inflammatory pathways. This highlights a promising strategy for the structural refinement and enhancement of phenolic acids to bolster their therapeutic outcomes.

While advancements have been noted in the research of MEHP phenolic acids, several hurdles remain for their clinical utilization. A significant challenge is pinpointing the dosage that is both efficacious and safe, given the potential for toxicity at elevated levels. Moreover, the interplay between phenolic acids and other medications could potentially influence their therapeutic efficacy. Furthermore, variability across batches of MEHP phenolic acids demands stringent standardization and quality control measures. Additionally, the long-term safety and any adverse effects of phenolic acids are subjects that warrant further investigation. Lastly, there is an evident need for more clinical trials to substantiate the therapeutic efficacy and safety of phenolic acids in managing inflammatory conditions.

To meaningfully tackle these challenges, considerable research on MEHP phenolic acids remains to be conducted. Firstly, delving into the correlation between the molecular structure of phenolic acids and their biological activity is essential, enabling the design and development of more potent phenolic acid derivatives. Secondly, the innovation of drug delivery systems should be prioritized to enhance the bioavailability and stability of these compounds. Personalization of phenolic acid therapy, tailored to an individual’s genetic and metabolic profile, presents a promising avenue for exploration. Moreover, investigating the synergistic use of phenolic acids with other pharmaceuticals or therapeutic approaches could potentially amplify their therapeutic impact. Notably, unraveling the intricate molecular mechanisms of phenolic acids, particularly their influence on cellular signaling pathways, is also a critical area for further research. Lastly, an increased number of clinical trials are imperative to yield conclusive evidence regarding the efficacy and safety of phenolic acids in combating inflammatory diseases.

In essence, MEHP phenolic acids possess significant commercial potential as both “anti-inflammatory drugs” and “ anti-inflammatory functional foods,” thereby fostering a healthier future for all.

## Author contributions

JX: Writing – original draft. SX: Writing – original draft. YL: Writing – review & editing. BX: Writing – review & editing. ML: Writing – review & editing. ZZ: Writing – review & editing. ZS: Writing – review & editing. QP: Writing – review & editing. CL: Writing – review & editing. DL: Conceptualization, Project administration, Writing – review & editing. LL: Conceptualization, Project administration, Writing – review & editing.
